# MERS-CoV at the Animal–Human Interface: Inputs on Exposure Pathways from an Expert-Opinion Elicitation

**DOI:** 10.3389/fvets.2016.00088

**Published:** 2016-10-05

**Authors:** Anna L. Funk, Flavie Luce Goutard, Eve Miguel, Mathieu Bourgarel, Veronique Chevalier, Bernard Faye, J. S. Malik Peiris, Maria D. Van Kerkhove, Francois Louis Roger

**Affiliations:** ^1^UEME, Institut Pasteur, Paris, France; ^2^Cirad, UPR AGIRs Research Unit, Montpellier, France; ^3^UMR MIVEGEC, IRD 224-CNRS 5290-UM, Montpellier, France; ^4^HKU-Pasteur Research Pole, Hong Kong, China; ^5^School of Public Health, University of Hong Kong, Hong Kong, China; ^6^Center for Global Health, Institut Pasteur, Paris, France

**Keywords:** MERS-CoV, animal–human interface, transmission, epidemiology, infection, risk factors

## Abstract

Nearly 4 years after the first report of the emergence of Middle-East respiratory syndrome Coronavirus (MERS-CoV) and nearly 1800 human cases later, the ecology of MERS-CoV, its epidemiology, and more than risk factors of MERS-CoV transmission between camels are poorly understood. Knowledge about the pathways and mechanisms of transmission from animals to humans is limited; as of yet, transmission risks have not been quantified. Moreover the divergent sanitary situations and exposures to animals among populations in the Arabian Peninsula, where human primary cases appear to dominate, vs. other regions in the Middle East and Africa, with no reported human clinical cases and where the virus has been detected only in dromedaries, represents huge scientific and health challenges. Here, we have used expert-opinion elicitation in order to obtain ideas on relative importance of MERS-CoV risk factors and estimates of transmission risks from various types of contact between humans and dromedaries. Fourteen experts with diverse and extensive experience in MERS-CoV relevant fields were enrolled and completed an online questionnaire that examined pathways based on several scenarios, e.g., camels–camels, camels–human, bats/other species to camels/humans, and the role of diverse biological substances (milk, urine, etc.) and potential fomites. Experts believed that dromedary camels play the largest role in MERS-CoV infection of other dromedaries; however, they also indicated a significant influence of the season (i.e. calving or weaning periods) on transmission risk. All experts thought that MERS-CoV-infected dromedaries and asymptomatic humans play the most important role in infection of humans, with bats and other species presenting a possible, but yet undefined, risk. Direct and indirect contact of humans with dromedary camels were identified as the most risky types of contact, when compared to consumption of various camel products, with estimated “most likely” incidence risks of at least 22 and 13% for direct and indirect contact, respectively. The results of our study are consistent with available, yet very limited, published data regarding the potential pathways of transmission of MERS-CoV at the animal–human interface. These results identify key knowledge gaps and highlight the need for more comprehensive, yet focused research to be conducted to better understand transmission between dromedaries and humans.

## Introduction

Nearly 4 years after the first report of the emergence of Middle-East respiratory syndrome Coronavirus (MERS-CoV) in humans and more than 1800 human cases later (1), mainly in Saudi Arabia (~75% of cases and almost all of the primary cases), the ecology of MERS-CoV and its epidemiology remain poorly understood (2). Human-to-human transmission of MERS-CoV accounts for approximately half of all the MERS-CoV cases reported to date (2). Inter-human transmission has been well documented in health care-associated outbreaks in the Middle East and Korea (3–5), and there appears to be limited inter-human transmission in household settings (6).

**Figure 1:**
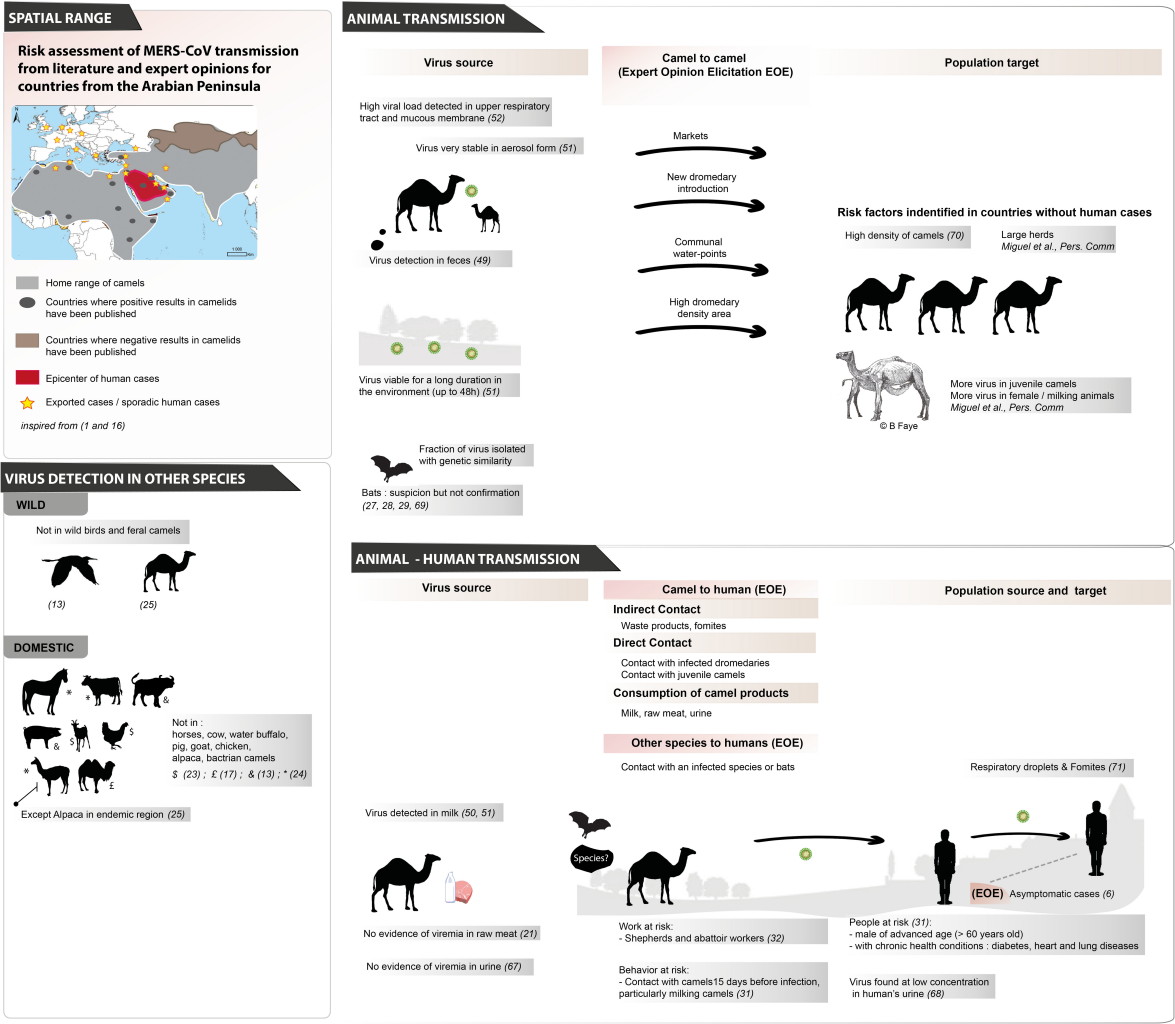
**Review of MERS-CoV exposure pathways for animal-to-animal transmission and animal-to-human transmission based on literature evidence and the expert opinions elicited in this study (67–71)**.

Many studies have now identified dromedary camels (*Camelus dromedarius;* dromedaries) as a natural host for MERS-CoV, and there appears to be ample evidence of widespread infection (either past or present) in dromedaries in the Middle East (7–10) and in many parts of Africa (11–15). High levels of MERS-CoV specific seroprevalence have been observed in dromedaries, ranging from 0% in Central Asia to as much as 100% in Africa and the Arabian Peninsula (7–17) (see Figure 1). MERS-CoV strains isolated from dromedaries are genetically and phenotypically very similar or identical to those infecting humans (18, 19).

Since the beginning of the MERS-CoV outbreak, animals and specifically dromedaries, have been suspected of playing a role in transmission. The global camel population has more than doubled in the past 50 years, reaching ~30 million today, 95% of which are dromedaries. Approximately 60% of camels are found in East African countries, which are important exporters to the Arabian peninsula and Egypt (20). Camels play a major role in socio-cultural traditions in Saudi Arabia; a place where the camel population has increased from 80,000 to 200,000 heads over the last 50 years; a number which some experts estimate is actually closer to 800,000 heads (21). In parallel, a drastic decrease (from 10 to 1.5%) of nomadic camel populations has been observed over this time period in favor of permanent (or semi-permanent) settlements often at the borders of cities (21). It is possible that the mentioned changes in global dromedary population dynamics have led to an increased spread and heightened detection of MERS-CoV in this species, both of which have made dromedaries the focus of most of the research conducted on MERS-CoV to date.

While coronaviruses are widespread in the animal kingdom (22), MERS-CoV seems to have a narrow host range. In the last few years, a large spectrum of domestic species have been negative after MERS-CoV serology tests, including horses, cattle, pig, water buffalo, chickens, goats, and Bactrian camels (13, 14, 17, 23, 24). An exception was published recently when antibodies were detected in Alpaca (*Vicugna pacos*) in Qatar; this is notably in a specific region where MERS-CoV is already endemic in dromedary camels (25) (Figure 1).

A number of studies on wild birds and swine in Hong Kong, feral camels in Australia and bats in several countries have not identified MERS-CoV in these species (13, 26) (Figure 1). Putative precursors of MERS-CoV have been detected in species of African bats (27), and Corman and colleagues raised hypotheses on the emergence of MERS-CoV from other animal species (28). They characterized the full genome of an African bat virus closely related to MERS-CoV and showed that human, camel, and bat viruses have phylogenetic relationships although these bat viruses are not closely similar to MERS-CoV. They suggest that, according to available serologic data on camels and humans since 2012 and molecular investigations of known cases, MERS-CoV moved from bats to camels in sub-Saharan Africa. They also suggest that camelids could be “mixing vessels for MERS-CoV and other mammalian CoVs” and that the virus can be transmitted between humans and camels (28). Up to now, MERS-CoV-like viruses have not been detected in any species other than camels, with the exception of an unconfirmed report of the detection of a very small fragment of MERS-CoV-like RNA in a specimen from a *Taphozous perforatus* bat collected in Saudi Arabia (29). *T. perforatus* and other bat species sampled in Egypt and Lebanon did not reveal MERS-CoV like viruses, although other coronaviruses were detected (30).

However, after more than 1800 reported cases over the past 4 years from 27 countries, only one case–control study evaluating non-human risk factors for infection has been performed and published (31). This study, which included 30 primary cases and 116 age, sex, and neighborhood-matched controls, confirmed suspicions that direct and indirect exposure to dromedary camels in the 14 days prior to symptom onset are risk factors for infection (31). This study also found that advanced age (>60 years old), being male, and having certain underlying chronic health conditions, such as diabetes, heart conditions, and chronic lung disease, were independent risk factors for disease (31). Several other seroprevalence studies evaluating the extent of MERS-CoV infection in occupationally exposed persons (e.g., farmers, herders, slaughterhouse workers) have identified that these populations have a higher levels of seroprevalence (32, 33) when compared to the general population (32) (see Figure 1).

What is currently unclear is why all primary human MERS-CoV cases have been reported from the Arabian Peninsula (2). Given that there is evidence of MERS-CoV circulation in dromedaries across large parts of Africa (11–15), it is likely that cases of MERS-CoV in humans have been missed. There are several potential hypotheses to explain this. First, surveillance for MERS-CoV in human populations focuses mostly on severe disease and on travelers returning from the Arabian Peninsula rather than on patients without a history of travel. Moreover, on-going surveillance in Saudi Arabia is now very intensive (34). Second, the prevalence of chronic underlying medical conditions in many countries in Africa is far lower than in the Middle East, with high rates of heart disease, diabetes, and obesity; third, it is likely that asymptomatic, mild or sub-clinical cases are missed with even the most robust surveillance systems. Fourth, the nature of contact with and the use of dromedary products differ between countries and cultures. Lastly, the viruses circulating in the Arabian Peninsula may be different. Although MERS-CoV in Africa are >99% identical at the nucleotide level with those in the Arabian Peninsula (35), it is conceivable that a few key amino-acid differences may make a major change in transmissibility and virulence.

In the case of MERS-CoV transmission, there is a large uncertainty about the various exposure pathways associated with new dromedary camel or human cases, and, although published research on MERS-CoV is actively increasing (36), few transmission risks have yet been quantified. There is an obvious need to collect more critical information from virological and eco-epidemiological studies, but also from social sciences (anthropology, sociology) studies about camel–human relationships, including behaviors at the interface. These studies can evaluate contact patterns, modes of transmission, viral shedding from animals, virus persistence in different environments, and biological samples. In view of all that remains to be done, we advocate a risk-ranking approach based on exposure pathways to guide allocation of resources for future data collection on the main sources of transmission of MERS-Cov. Risk assessment is a powerful modeling tool that enables decision-makers to determine the likelihood of disease occurrence and the magnitude of its consequences, which, in turn, allows identification of key steps and appropriate management measures to take. It is a structured and a systematic process that helps in the gathering of diverse and disparate information and data. However, when data are scarce and knowledge gaps are highly prevalent, such as with the recently identified MERS-CoV, too many transmission pathways would have been presented for the risk analysis. This is why we proposed, as a preliminary step, to call upon experts using expert-opinion elicitation (EOE), to explore scenarios and hypotheses of transmission among animal(s), fomites, and humans. From the EOE outputs, a qualitative and/or quantitative risk assessment model could then be implemented. Expert-opinion elicitation has proven to be useful in other zoonotic disease risk assessments, especially in cases where little quantitative information for the disease is already known (37, 38). The aim of this work is to allow for a triage of highly likely and unlikely pathways, and highlight areas that deserve increased attention for field surveys and studies.

## Materials and Methods

In our study, experts were defined as being persons with relevant experience on the topic, including having extensive technical experience in epidemiological or virological research through MERS-CoV or related animal and/or human studies. Considering the recent emergence of the virus as a cause for human disease, extensive experience in MERS-CoV research itself was not an inclusion criteria; however, all included experts needed to have some experience working on MERS-CoV and/or camel research topics within North Africa and the Arabian Peninsula, if not elsewhere. Furthermore, the experts’ publications and professional affiliations should have been significant enough to reflect this expertise. Recruitment was done first through relevance screening, where the researchers chose persons based on their own judgment. Following this original recruitment, “snowball” recruitment was used; experts who chose to participate were asked to recommend other experts to fill out the questionnaire. We aimed to enroll at least 10 experts, with extensive experience in relevant fields, for the exercise and, therefore, started by emailing invitations to 13 persons. All persons recommended by the first group of experts were invited to participate if their expertise was judged relevant for our study. All experts gave an informed consent before starting their participation in the survey. Written consent was not necessary for this type of study; experts could withdraw themselves from the study at any time and all opinion “results” would be presented in an anonymous fashion.

Searching into MERS-CoV literature (36, 39) and meeting reports (40) allowed us to identify potential pathways and risk factors needed for designing the EOE (see Figure 1).

The questionnaire was designed online using the tool Survey Monkey (www.surveymonkey.com). A pilot survey was sent to the team members in order to test the survey and optimize the consistency of the questions. A clear description of the study objectives and of what was expected for their participation was provided to experts in the invitation email. Following their acceptance to participate, the experts were emailed a link to the online survey. The beginning of the online survey included instructions, examples, and contact information of the administering researchers.

The questionnaire was designed to take about 30–40 min, and be filled in by experts individually using a link to online software (see Image S1 in Supplementary Material). It consisted of expertise questions, relative importance of risk factor questions, transmission risk estimations, and open-ended responses, in that order, all of which will be described in more detail below.

**Figure 2:**
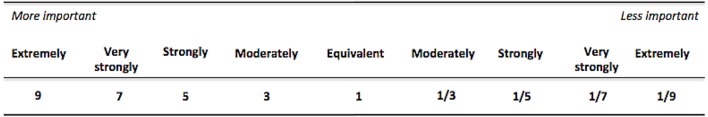
**Simplified Saaty Scale used for comparing risk factors in the analytical hierarchy process**.

The analytical hierarchy process (AHP) (41, 42) is widely used in marketing research (43) and has more recently been introduced as a tool in veterinary epidemiology (38, 44). AHP obtains opinions on the weight of the relative importance of one attribute of an object or event over another, through pair-wise comparisons. In our questionnaire, we used the technique to obtain experts opinions about the most relevant exposure pathways and their relative importance for five different animal–animal or animal–human transmission scenarios. Where appropriate, simple transmission diagrams were used to explain the potential exposure pathways in question. Experts were first asked to identify which exposure pathway they “believed in” out of a provided list, always with the opportunity to specify “other.” They were then asked to do pair-wise comparisons of each exposure pathway, comparing the risk factors of transmission, using the Saaty scale (41) (Figure 2).

In order to obtain quantitative estimates on the transmission risks from dromedaries to humans, we asked experts for their 3-point estimation (minimum, most likely, and maximum) considering different types of exposure between 10 susceptible camel workers and dromedaries. Exposures included consumption of camel products (e.g., milk, urine, meat), direct and indirect contact; separate estimations were asked for different scenarios of younger (≤50 years) or older (>50 years) camel workers and adult or juvenile dromedaries. Using the same method, experts were also asked their estimates for transmission between potentially asymptomatic camel workers and family contacts.

Finally, a few open-ended questions on factors that may increase or decrease transmission and were posed to experts.

The survey was not anonymous in order to be able to come back to the experts in case of inconsistency in their answers. For every question, the experts were asked to respond not only expressing their opinion but also to assess their own confidence in their answers for each question, with a score from 1 to 5.

The analytical hierarchy process allowed us to weight each exposure pathway according to the level of importance given to it by each expert. Additional weight was attributed to each answer according to the level of confidence given by the expert. Then, for each pathway, a weighted aggregation of all expert answers was generated. An expert’s data were excluded from the combined estimates in case of any of the following criteria: <30% consistency ratio, obvious erroneous entry, missing data for part of or the entire question. In this case, a 30% consistency ratio cut-off, taking into consideration that the historically recommended 10% cut-off is shown to be too severe for comparison matrices that have >3 variables and that the cut-off should increase with number of variables (45, 46); our questions introduced up to eight variables for each matrix. The overall level of agreement across experts in their ranking of the selected risk factors was calculated using the Kendall’s W coefficient. The average weighted minimum, most likely and maximum transmission risk for each of the 3-point estimation questions was also generated using a similar weighting mechanism. An expert’s data was excluded from the 3-point distribution combined estimates in case of consistently highly outlying estimates or missing data for part of or the entire distribution. Outliers were defined as estimated risks that were consistently greater than twice the estimates of all other experts. When possible, for missing or erroneous data, experts were re-contacted by email to clarify. The mean confidence level, across all included experts, was calculated for each pair-wise comparison and 3-point distribution question. Open-ended responses were summarized qualitatively.

## Results

Overall, 18 experts were contacted to take part in the questionnaire. Of these, 16 responded to the invitation, and 14 filled out the questionnaire in full, contributing data to this study. All respondents, except 1, had expertise in either MERS-CoV epidemiology and/or virology; the remaining expert had significant experience in camel production and husbandry and general epidemiology. Six and three respondents had experience in conducting studies of camels and bats, respectively. A detailed description of each participant’s expertise can be found in Table 1.

**Table 1:** **Included Expert Profiles**.

	Degree	Epidemiology		Virology		Camel studies		Risk analysis		Chiropterology (bats)	
1	MD	✓^a^	1–5 years	✓^a^	1–5 years						
2	DVM	✓^a^	10+ years	✓^a^	10+ years	✓	10+ years				
3	MD	✓^a^	10+ years	✓^a^	10+ years						
4	MPH	✓^a^	6–10 years			✓	1–5 years	✓	1–5 years		
5	PhD	✓^a^	10+ years	✓	1–5 years			✓	6–10 years		
6	DVM	✓^a^	10+ years	✓^a^	10+ years	✓	10+ years	✓	6–10 years	✓	10+ years
7	DVM	✓	10+ years	✓^a^	10+ years						
8	DVM	✓^a^	1–5 years								
9	PhD	✓^a^	1–5 years	✓^a^	6–10 years			✓	10+ years		
10	DVM	✓	10+ years			✓	10+ years				
11	DVM	✓^a^	10+ years					✓	6–10 years		
12	PhD	✓^a^	1–5 years			✓	1–5 years	✓	1–5 years		
13	MD	✓^a^	6–10 years	✓^a^	10+ years					✓	6–10 years
14	DVM	✓^a^	10+ years	✓^a^	10+ years	✓	1–5 years	✓	6–10 years	✓	1–5 years

*^a^Including MERS-CoV specific*.

### MERS-CoV Infection of Dromedary Camels

**Figure 3:**
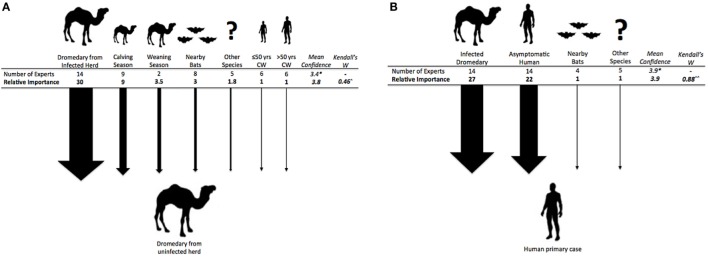
**(A)** (left). **Exposure pathways and relative weights of risk factors for a dromedary camel from an uninfected herd to become infected with MERS-CoV**. ^^^*p* < 0.001. **(B)** (right). **Exposure pathways and relative weights of risk factors for a camel worker or other human to become infected with MERS-CoV**. ^^^^*p* < 0.01. *Mean confidence for overall choice of risk factors for this question with a scale of confidence between 1 (not confident) and 5 (very confident).

On the topic of how dromedaries become infected with MERS-CoV, the following exposure pathways were presented to experts: infestation of infected bats in close proximity, daily close contact with infected camel workers (both ≤50 and >50 years old), short-term contact with an infected dromedary herd, short-term contact with a non-dromedary species infected with MERS-CoV, and infection occurring during dromedary calving season. All of the above risk factors were selected by at least 5/14 experts. However, the most highly selected and importantly weighted exposures were “short-term contact with an infected dromedary herd” and “timing coinciding with dromedary calving season” (Figure 3A). Two experts selected the “other” option and specified the most risky season is dromedary-weaning season. Furthermore, one expert selected “other” and included the possible risk associated with contaminated camel feed.

### MERS-CoV Infection between Dromedary Herds

Risk factors that were presented to experts, when asking about the possibility of dromedaries from different herds infecting each other, were: nomadic dromedary herds, introduction of a new dromedary into the herd, high dromedary density area, dromedaries taken to racetracks, dromedaries entered into/taken to beauty contests, dromedaries brought to communal water-points, dromedaries brought to/sold at markets, dromedaries pass through border points. Each factor was considered risky by at least 5 (36%) of the 14 experts, and no additional risk factors from experts were provided. The most highly selected (i.e., >70% of experts) risk factors, in order of weighted importance, were: bringing dromedaries to markets, introduction of a new dromedary into the herd, high dromedary density area, and bringing dromedaries to communal water-points. The first three of these risk factors were given similar weights by experts, while the last (communal water-points) was thought to only be half as important as them. Experts had sufficient agreement on their ranking of risk factors (Kendalls W = 0.25, *p* = 0.003), and had a mean response certainty of 3.9 and 3.8 (out of 5) for choosing risk factors and the subsequent rankings, respectively.

### MERS-CoV Primary Infection in Humans

Exposure pathways for human primary case occurrence included: infestation of MERS-CoV infected bats in close proximity to human populations, contact with a MERS-CoV infected herd of dromedaries, contact with a non-dromedary MERS-CoV infected species, blood-biting pests (e.g., fleas, ticks) on an infected animal species or on humans, contact with another human who is asymptomatically infected with MERS-CoV. All experts agreed that contact with infected dromedaries or asymptomatic humans were major risks for disease transmission, with the former being of higher risk (Figure 3B). About a third of experts (29–36%) thought that contact with MERS-CoV infected species other than dromedaries or bats may also play a role in human infection. Only one expert considered the possibility of blood-biting pests transmitting infection between dromedaries or other species and humans. Experts suggested no “other” risk factors.

### Transmission from Infected Dromedaries to Camel Workers

**Figure 4:**
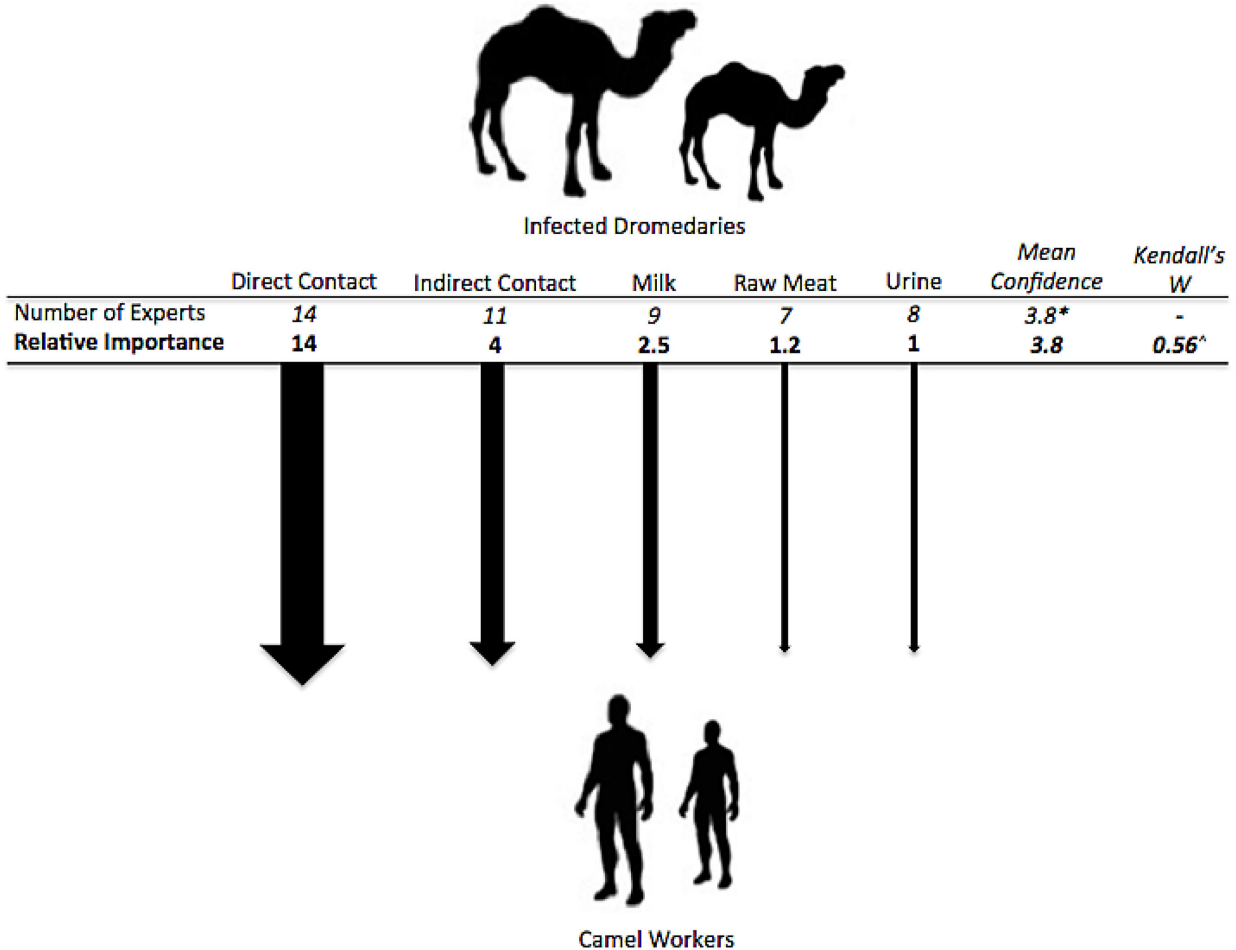
**Exposure pathways and relative weights of risk factors for types of transmission between dromedaries and camel workers**. ^^^*p* < 0.01. *Mean confidence for overall choice of risk factors for this question with a scale of confidence between 1 (not confident) and 5 (very confident).

The following possible exposure pathways from dromedaries to camel workers were presented to the experts: direct contact (e.g., face-to-face, touching, kissing), indirect contact (e.g., cleaning camel environment, contact with dromedary waste), consumption of unpasteurized milk, consumption or use of dromedary urine, and consumption of raw dromedary meat. All pathways were thought to be possible by the experts (≥50% each) and direct contact with dromedaries was thought to be a transmission risk factor by all experts (Figure 4). When asked to quantify these risks, by estimating the likely incidence of human cases, separately for adult and juvenile camels and older (>50 years) and younger (≤50 years) camel workers, experts estimated direct and then indirect contact with the highest risk; generally there was a slightly higher risk estimated when contact was with juvenile camels, and a clear trend for higher estimated risk when older vs. younger camel workers were exposed (see Table 2). Specifically, the risk of transmission was thought to be low (≤5%) for camel workers consuming or using camel products, such as milk, urine, or raw meat. The estimation of the incidence was quantified as being between 13 and 24% for indirect contact with an infected dromedary regardless of whether adult or juvenile, and between 22 and 33% with direct contact, varying by the age of both the camels and camel workers.

**Table 2:** **Estimated percentage transmission risk from adult and juvenile dromedaries to camel workers (CW)**.

	Adult dromedary						Juvenile dromedary					
	≤50-year-old CW			>50-year-old CW			≤50-year-old CW			>50-year-old CW		
	Most likely	Min/max	C^a^	Most likely	Min/max	C^a^	Most likely	Min/max	C^a^	Most likely	Min/max	C^a^
Milk	3	0/13	2.9	4	1/16	2.8	–	–	–	–	-	
Urine	3	0/9	3.2	3	0/12	2.9	5	0/12	3	4	0/12	2.9
Raw meat	4	0/15	2.8	3	0/13	2.9	1	1/6	2.9	5	2/14	2.8
Direct contact	25	4/45	2.9	29	5/55	3	22	7/39	2.9	33	8/57	3.2
Indirect contact	13	1/33	2.9	18	4/36	2.8	19	4/34	3.1	24	6/48	3.1

*^a^C = Mean level of expert confidence for estimate with a scale of confidence between 1 (not confident) and 5 (very confident)*.

### Transmission from Asymptomatic MERS-CoV Cases to Contacts

Experts were asked to estimate the risk of transmission from an asymptomatic infected individual to other individuals in close contact. The estimated “most likely” risk of transmission if the potentially asymptomatic camel workers were either ≤50 years or >50 years old was 9%. The experts had a mean confidence of 2.8 (out of 5) for their answers for both age groups.

### Risk Factors for Symptomatic MERS-CoV Infection in Humans

At least 10 of the 14 experts agreed that older age (>50 years), being immunocompromised, and the amount of viral dose transmitted, increases the chances that infected camel workers or other persons will become symptomatic after MERS-CoV infection. Being immunocompromised was given the highest overall comparative weight as a risk factor, followed by amount of viral dose transmitted. Also, genetic susceptibility and recent occurrence of an epidemic period for another disease (e.g., Influenza) were identified as risky by three and four experts, respectively. Experts had a good level of agreement on their ranking of the selected risk factors (Kendalls W = 0.61, *p* < 0.01), and had a mean response certainty of 3.4 and 3.3 (out of 5) for choosing risk factors and the subsequent rankings, respectively.

### Responses to Open-Ended Questions on Transmission Dynamics

Experts were asked which factors led to increase viral shedding in MERS-CoV infected dromedaries. The most highly suggested items included: juvenile dromedaries lacking antibody immunity (*n* = 4), immunosuppressive conditions and secondary disease (*n* = 4), animal density (*n* = 2) and stressful environments for the animals (e.g., at slaughterhouses, markets) (*n* = 2). Experts believed factors that may lead to increased or more efficient transmission between MERS-CoV infected dromedaries and humans include repeated close contact with dromedaries with the chance of contact with respiratory secretions (*n* = 3), host susceptibility or immune status (*n* = 2), increased virulence of the virus through genetic recombination or other (*n* = 2), and environmental contamination of camel-visited areas (*n* = 2). Experts were also asked whether or not they thought any other viruses might cross-immunize with MERS-CoV for either dromedaries or humans. Of the 12 experts who answered this question, 3 said “No,” while 6 were unsure or thought this was possible, and 3 experts believed that other coronaviruses might cross-immunize with MERS-CoV.

## Discussion

Our results use expert opinion to weigh the different transmission risks of MERS-CoV between animals and from animals to humans. Despite a lack of quantitative data, our results are supported by growing evidence from research published from MERS-CoV affected countries. Risk assessment is a tool that allows for the gathering of accessible data and information (e.g., expert opinion). The preliminary approach proposed in this paper synthesized available evidence regarding primary MERS-CoV transmission to humans. Our results highlight a general consensus in the order/rank of pathways, as well as for potential drivers and risk factors. According to the experts included in our study, dromedaries play a major role in transmission. However, the role of bats could not be ruled out and should be investigated further.

Despite the fact that new research reveals traces of antibodies against MERS-CoV in two livestock handlers in Kenya in 2013/14 (47), it is surprising that no locally acquired primary human cases have been reported where humans and infected dromedary camels are present outside the Arabian Peninsula. Recent workshops on MERS-CoV (Doha in April 2015 and Cairo in May 2015), organized by WHO, FAO, and the World Organisation for Animal Health (OIE), have produced numerous recommendations to improve surveillance and suggest research in animal and human populations (48). One of these recommendations is to investigate whether and why MERS-CoV infections of humans appear not to occur in Africa despite the high levels of infection in dromedaries, and why the virus is apparently absent in camels in Central Asia (dromedary and Bactrian camels).

The exact role of dromedary camels as a potential reservoir for MERS-CoV is also still unclear, and further investigations should be carried out to identify the mechanism of virus transmission and quantify the stability of the virus in various conditions more clearly. MERS-CoV has been detected in the oropharyngeal tract, feces, milk, and meat of dromedaries (21, 49–51). However, the modes of transmission are not clearly known. Our experts felt that the transmission risk from consumption of raw camel products, including milk, meat, and urine is low. It is assumed that the infection from dromedary camels to humans occurs through droplets or contact as high viral loads have been detected in the upper respiratory tract and nasal mucous membrane of dromedaries (52). However, milking activities and drinking unpasteurized milk, which is highly prevalent in Saudi Arabia (53), are considered as risky for the occurrence of primary cases in human populations. There is no evidence of MERS-CoV in camel meat, and it is known that cooking would kill the virus. One study from Qatar identified MERS-CoV in milk, but it was unclear whether the virus was excreted in the milk or if the milk had been contaminated by traditional milking techniques, which involves calves being used to initiate the milking process (50).

The role of and the extent to which infected asymptomatic human cases play a role in transmission is unknown. WHO estimates that ~20% of reported MERS cases are asymptomatic (54), but this estimate is likely underestimated given surveillance focuses on severe cases requiring hospitalization (55) and evidence from serologic studies (32). One study documented prolonged shedding of MERS-CoV in an asymptomatic health care worker (56), which provides evidence that, if not properly isolated, asymptomatic cases in health care settings and in the community could lead to onward transmission. The experts included in this study believe that contact with asymptomatic cases is as important as that with infected dromedaries. Comprehensive testing of contacts of MERS-CoV patients, regardless of the presence of symptoms, is required to evaluate infection between known cases. The role of asymptomatic cases or carriers, if they are indeed infected, also needs careful consideration in the community setting. Not all reported primary cases can be traced back to contact with dromedaries, and it is likely, at least in some cases, that an asymptomatic or mildly symptomatic case may be an intermediary between dromedary contact and a symptomatic human case.

After 4 years, research on the role of camels and/or other sources of primary transmissions to human is inadequate. So far, most MERS-CoV studies have focused on virological or clinical aspects of the disease. No comprehensive analytical epidemiological studies have yet been carried out in MERS-CoV affected countries. With the exception of one case–control study (31) and individual case studies following investigation into single cases, transmission between dromedary herds and between dromedaries and humans has not been well studied. Even these detailed investigations are limited in terms of deciphering the cause-effect relationship. As human cases of MERS are relatively sporadic/rare, case control studies, especially matched case–control studies, can be well adapted during epidemics or outbreaks investigations and must be performed. Cohort studies are the best option in order to compare incidence among exposed (e.g., camel workers, immunocompromised people, etc.) and non-exposed populations. However, conducting cohort studies for rare diseases may be difficult.

In regions without reported human clinical cases of MERS, cross-sectional surveys based on serological investigation in humans and identification and quantification of potential risk factors for infection (behaviors, husbandry, contacts with camels and camel products, etc.) will assist in the suggestion of hypotheses, if human infection is prevalent and statistically exploitable for inference at the population level. Outside of the Middle East, these studies need to be undertaken, especially outside of the Arabian Peninsula and in African countries where MERS-CoV has been detected and/or isolated in dromedaries. Outside of the Arabian Peninsula, a number of joint human/camel serological studies are currently underway in North Africa (Pasteur Institut, Pers Comm) and planned in sub-Saharan African countries (HKU and Cirad, Pers Comm). For instance, in Ethiopia where MERS-CoV strains have been detected in camels (Peiris et al., Pers Comm), studies in at-risk human communities (e.g., nomadic people in close contact with camels, abattoir workers) have to be implemented: both analytical epidemiological studies and surveys on acute febrile illness (57), including respiratory and other signs could lead to clues about MERS-CoV infection and/or MERS-disease in humans.

At a more global scale, understanding differences in exposures and behaviors of individuals with dromedaries across the Middle East and Africa is likely to explain some of the differences in potential infection risk. For that purpose, “ecological studies” could help to explore diverse drivers of transmission among different environments and societies. However, studies based on aggregated data are prone to many biases (58) making it difficult to know if individuals have really been exposed to the risk factor in question. Furthermore, social sciences have to be enlisted in order to puzzle out the relationships between camels and humans. Outputs can serve for epidemiological studies and modeling (e.g., multi-agents systems, see hereunder). Additionally, improved surveillance systems in humans and animals in rural and nomadic areas are required for MERS-CoV considering possible changes of the public health situation due to virus evolution (e.g., toward more pathogenic strains or diffusion of strains from areas with human disease, etc.) over time, modification of camel husbandry, etc. For population-based studies, epidemiology and surveillance, we need to have species-adapted and validated serological tools. Indeed, performances of tests are often lacking and should be assessed using frequentist or Bayesian approaches.

In addition to epidemiological studies, additional data from viral ecology studies among camels and other species, including bats, are required; phylogeography studies of MERS-CoV, and ecological studies on bat species living in the proximity of camels and suspected to play a role in the circulation of the virus, including a better understanding of their home ranges, migration patterns, biology (especially reproduction), roosting sites, and mechanisms of contact with camels are needed. Studies of viral shedding in animals, of virus persistence in different biological specimens of humans and animals, and in the environment under different conditions would help to quantify, or at least help to characterize, potential transmission risks.

The effect of MERS-CoV on camel health is not well documented; is the camel an asymptomatic carrier (reservoir/vector) or can MERS-CoV infection induce mild symptoms and/or pave the way for secondary infections? To address this question, camel studies should focus not only on MERS but also on the diverse etiologies of respiratory syndromes (59). This could be significant because, first, if MERS is recognized as a camel disease, more research resources could be allocated, second, super-infections could play a role in MERS-CoV transmission traits. Finally, multi-pathogens studies and multi-disease surveillance in camel populations can improve, through an economy of scale, MERS-CoV detection and the collection of data and metadata. Similarly, health conditions and infectious and parasitic diseases of camels may have an impact on MERS-CoV ecology and/or MERS epidemiological features. Indeed, immunosuppressive effects of several origins (husbandry and farming conditions, under-nutrition, deficiencies, parasites, co-infections, etc.) could enhance the infectivity of the MERS-CoV.

Considering the recent emergence of MERS-CoV as a zoonotic threat, and the lack of information already quantified on it, we appropriately included a small number of experts in this EOE exercise, but those who had diverse and extensive experience in relevant fields. The questions included in the exercise were feasible for persons who are not accustomed to formal prioritization methods; AHP is known to be adapted for complex information situations, to be intuitively understandable and to allow scientists to score the attributes with minimal confusion. Our study has several limitations. First, it is commonly considered best practice to give a training exercise on EOE methods as well as provide a multi-page literature review on the topic in question to participating experts, prior to administering the questionnaire, however, this was not done here. In this case, experts were provided with a detailed document describing how to fill out the questionnaire, with examples, and were invited to contact the authors if they had any questions or confusion (see Image S1 in Supplementary Material). It is possible to “calibrate” experts, by including some items in the questionnaire for which a general scientific consensus or quantification already exists; the expert response to these questions can then be matched to the real answer in order to see how close that expert arrives. There was no calibration done in this study, largely due to the fact that there are almost no solidly quantified risks associated with MERS-CoV at present. Experts were weighted instead only on their confidence level for each question answered; however, it is always possible that experts are overconfident, giving scores that do not reflect their real uncertainty on their knowledge of a variable. Linguistic uncertainty in the questionnaire could have led to some bias; experts with varied origins and experience can interpret questions and imagine contexts differently, and this can be exacerbated by ambiguity or lack of specificity in questions. For the aggregation of our results, we used a mathematical approach by combining the weighted estimates of all experts. Another option would have been to use a more inclusive and participatory behavioral approach that would allow experts to revise their answers after seeing those of others and eventually come to a consensus together. However, empirical results have suggested that mathematical methods can outperform behavioral techniques in certain circumstances (60, 61), and it is also possible that group dynamics could bias estimations of risks toward a more extreme consensus (62). Overall, the experts were more certain in answering the AHP questions, which involved choosing and ranking risk factors. For all animal–animal or animal–human AHP questions, the experts consistently had mean confidence levels of close to 4 (out of 5), however, when asked to rank risk factors for asymptomatic human–human transmission, the mean confidence level was lower (closer to 3). When estimating minimum, most likely, and maximum transmission risks based on scenarios, the experts had lower overall mean confidence in their answers, with scores of between 2.8 and 3.2 for all estimations. This lower certainty is likely related to the fact that so few transmission risks for MERS have yet been quantified, whereas in choosing and ranking risk factors, there are already strong trends as presented in the literature.

Apart from virological, ecological, and epidemiological approaches, simulation models will allow for the testing of different scenarios of transmission, and this can be compared with reported cases. However, the scarcity of the data at the animal–human interface impedes the use of data-driven models like the stratified (animal–human) SEIR models, contact networks models, etc. Moreover, in order to analyze and simulate the complete pattern of the disease, there is also a need to capture the behaviors of animals and people (63). The individual-based model built on multi-agents systems is a computerized system combining multiple interacting agents (e.g., humans, animals) within a given environment (64). Such a model could be built in close interaction with stakeholders (farmers, camel workers, etc. (65)) and could drive toward more precise hypotheses about initial transmissions to humans (66).

This EOE study has several limitations but it is a preliminary step for implementing a more comprehensive risk assessment. Risk assessment is a time-consuming and iterative process that needs to be fed by several sources of data, lab experiments and field observations (see Table 3 for a summary of recommended studies). Risk communication, which is part of the risk analysis and closely linked to the risk assessment, is essential, especially considering that MERS is a major public health issue and could have indirect economic and social impacts on the “dromedary world.” The questionable responsibility of dromedaries regarding human MERS-CoV cases could indeed spur inappropriate and overdone control measures. More broadly, this EOE can help in identifying gaps and needs in terms of experimental, field and modeling studies that will give a better understanding of the zoonotic transmission pathways of MERS.

**Table 3:** **Recommended MERS-CoV studies at the animal–human interface**.

Studies	Main outputs	Key strengths	Shortcomings and constraints
**1. Experimental studies**			
1.1 Virology	Virus strains comparisons among animals and humans. Phylogeography	Deciphering of pathways between mammals species	Statistical power: require sufficient and representative strains to be analyzed
1.2 Experimental infections in bats and camels (and other livestock species)	Pathophysiology and clinical outcomes. Immunological response. Virus ecology; virus shedding in animals	Epidemiological parameters for modeling, e.g., shedding, viral excretion	Bioethics. Biosecurity. Costly
**2. (Empirical) Observational studies**			
2.1 Ecological studies on bats and camels	Roles as reservoirs and/or vectors of MERS-CoV	Identification of drivers of MERS-CoV ecology	Authorization to work on endangered bats. Need efficient non-invasive methods. Devices to follow livestock movements and bats migrations
2.2 Epidemiological studies	Prevalence and incidence in camels/humans. Serological test performance in humans/animals. At-risk behaviors and risk factors for MERS-disease in humans	Cross-sectional and ecological studies, which are relatively simple to be carried out	Costly for case–control and cohort studies
2.3 Sociology and anthropology studies	At-risk human behaviors at individual and community levels	Will feed epidemiological studies and models	Implementation of participatory approaches in pastoral and challenging territories (e.g., low-income countries, remote areas)
2.4 One health surveillance systems	Follow-up of virus, antibodies, clinical signs in humans and animals	Detection of emergence in humans; collection of viruses. infection timeline	Complex (need agreement among public health and vet services) and costly (need sustainability)
**3. Modeling**			
3.1 Probabilistic models (e.g., QRA)	At-risk pathways of transmission	Useful for disease management even if all mechanisms are not known	Long and iterative process for QRA. Data and information needed, including experiment data
3.2 Dynamic models (e.g., SIR, IBM, SNA)	Testing hypotheses (simulation) of MERS-CoV transmission. Drawing up the levels of vaccination needed	Deciphering of transmission ways between mammals species	Need data. Complex models required (SIR stratification animal/human, joint models, e.g., SIR and SNA, etc.)
3.3 Multiple-criteria decision-making or MCDA	Decision process. Risk mapping for spatialized MCDA	Straightforward to be implemented (literature review and expert opinions)	Model validation (but could be done with Human cases in Arabian peninsula)

*SIR, compartmental models; IBM, individual-based modeling or multi-agent systems; SNA, social network analysis or contact network analysis; MCDA, multi-criteria decision analysis; QRA, quantitative risk assessment*.

## Author Contributions

MVK and FR designed the study, contributed to the analyses, and drafted the manuscript. AF and FG designed the data collection instrument, analyzed the data, and drafted the manuscript. MP, EM, MB, VC, and BF reviewed the results and drafted the manuscript. All authors agree with the conclusions of the paper.

## Conflict of Interest Statement

The authors declare that the research was conducted in the absence of any commercial or financial relationships that could be construed as a potential conflict of interest.

## References

[1] WHO. Middle East Respiratory Syndrome Coronavirus (MERS-CoV) – Saudi Arabia. (2016). Available from: http://www.who.int/csr/don/25-july-2016-mers-saudi-arabia/en/

[2] Embarek PKB Van Kerkhove MD Middle East respiratory syndrome coronavirus (MERS-CoV): current situation 3 years after the virus was first identified. Wkly Epidemiol Rec (2015) 90(20):245–50. 25980038

[3] Assiri A McGeer A Perl TM Price CS Al Rabeeah AA Cummings DA Hospital outbreak of Middle East respiratory syndrome coronavirus. N Engl J Med (2013) 369(5):407–16. 10.1056/NEJMoa1306742 23782161 PMC4029105

[4] Drosten C Muth D Corman VM Hussain R Al Masri M HajOmar W An observational, laboratory-based study of outbreaks of Middle East respiratory syndrome coronavirus in Jeddah and Riyadh, Kingdom of Saudi Arabia, 2014. Clin Infect Dis (2015) 60(3):369–77. 10.1093/cid/ciu812 25323704 PMC4303774

[5] Ki M. 2015 MERS outbreak in Korea: hospital-to-hospital transmission. Epidemiol Health (2015) 37:e2015033. 10.4178/epih/e2015033 26212508 PMC4533026

[6] Drosten C Meyer B Muller MA Corman VM Al-Masri M Hossain R Transmission of MERS-coronavirus in household contacts. N Engl J Med (2014) 371(9):828–35. 10.1056/NEJMoa1405858 25162889

[7] Azhar EI El-Kafrawy SA Farraj SA Hassan AM Al-Saeed MS Hashem AM Evidence for camel-to-human transmission of MERS coronavirus. N Engl J Med (2014) 370(26):2499–505. 10.1056/NEJMoa1401505 24896817

[8] Hemida MG Al-Naeem A Perera RA Chin AW Poon LL Peiris M Lack of middle East respiratory syndrome coronavirus transmission from infected camels. Emerg Infect Dis J (2015) 21(4):699–701. 10.3201/eid2104.141949 PMC4378477 25811546

[9] Hemida MG Perera RA Al Jassim RA Kayali G Siu LY Wang P Seroepidemiology of Middle East respiratory syndrome (MERS) coronavirus in Saudi Arabia (1993) and Australia (2014) and characterisation of assay specificity. Eurosurveillance (2014) 19(23):20828. 10.2807/1560-7917.ES2014.19.23.20828 24957744 PMC4674219

[10] Nowotny N Kolodziejek J. Middle East respiratory syndrome coronavirus (MERS-CoV) in dromedary camels, Oman, 2013. Eurosurveillance (2014) 19(16):20781. 10.2807/1560-7917.ES2014.19.16.20781 24786259

[11] Deem SL Fevre EM Kinnaird M Browne AS Muloi D Godeke G Serological evidence of MERS-CoV antibodies in dromedary camels (*Camelus dromedaries*) in Laikipia County, Kenya. PLoS One (2015) 10(10):e0140125. 10.1371/journal.pone.0140125 26473733 PMC4608777

[12] Müller MA Corman VM Jores J Meyer B Younan M Liljander A MERS coronavirus neutralizing antibodies in camels, Eastern Africa, 1983–1997. Emerg Infect Dis (2014) 20(12):2093–5. 10.3201/eid2012.141026 25425139 PMC4257824

[13] Perera RA Wang P Gomaa MR El-Shesheny R Kandeil A Bagato O Seroepidemiology for MERS coronavirus using microneutralisation and pseudoparticle virus neutralisation assays reveal a high prevalence of antibody in dromedary camels in Egypt, June 2013. Eurosurveillance (2013) 18(36):20574. 10.2807/1560-7917.ES2013.18.36.20574 24079378

[14] Reusken CB Haagmans BL Müller MA Gutierrez C Godeke G Meyer B Middle East respiratory syndrome coronavirus neutralising serum antibodies in dromedary camels: a comparative serological study. Lancet Infect Dis (2013) 13:859–66. 10.1016/S1473-3099(13)70164-6 23933067 PMC7106530

[15] Reusken CB Messadi L Feyisa A Ularamu H Godeke GJ Danmarwa A Geographic distribution of MERS coronavirus among dromedary camels, Africa. Emerg Infect Dis J (2014) 20(8):1370–4. 10.3201/eid2008.140590 25062254 PMC4111168

[16] Miguel E El Idrissi A Chevalier V Caron A Faye B Peiris M Ecological and epidemiological roles of camels: lessons from existing and emerging viral infections. FAO EMPRES Anim Health 360 (2016) 46:4–8. Rome.

[17] Miguel E Perera R Baubekova A Chevalier V Faye B Akhmetsadykov N Absence of middle east respiratory syndrome coronavirus in camelids, Kazakhstan, 2015. Emerg Infect Dis (2016) 22(3):555–7. 10.3201/eid2203.151284 26889787 PMC4766892

[18] Chan RWY Hemida MG Kayali G Chu DKW Poon LLM Alnaeem A Tropism and replication of Middle East respiratory syndrome coronavirus from dromedary camels in the human respiratory tract: an in-vitro and ex-vivo study. Lancet Respir Med (2014) 2(10):813–22. 10.1016/s2213-2600(14)70158-4 25174549 PMC7164818

[19] Farag EA Reusken CB Haagmans BL Mohran KA Raj VS Pas SD High proportion of MERS-CoV shedding dromedaries at slaughterhouse with a potential epidemiological link to human cases, Qatar 2014. Infect Ecol Epidemiol (2015) 5:28305. 10.3402/iee.v5.28305 26183160 PMC4505336

[20] Faye B Camel meat in the world. In: Kadim I Maghoub O Faye B Farouk M, editors. Camel Meat and Meat Products. Oxfordshire, UK: CABI (2013). p. 7–16.

[21] Gossner C Danielson N Gervelmeyer A Berthe F Faye B Kaasik Aaslav K Human-dromedary camel interactions and the risk of acquiring zoonotic Middle East Respiratory Syndrome coronavirus infection. Zoonoses Public Health (2014) 27(10):12171 10.1111/zph.12171 PMC7165574 25545147

[22] Woo PC Lau SK Lam CS Lau CC Tsang AK Lau JH Discovery of seven novel mammalian and avian coronaviruses in the genus deltacoronavirus supports bat coronaviruses as the gene source of alphacoronavirus and betacoronavirus and avian coronaviruses as the gene source of gammacoronavirus and deltacoronavirus. J Virol (2012) 86(7):3995–4008. 10.1128/JVI.06540-11 22278237 PMC3302495

[23] Hemida MG Perera RA Wang P Alhammadi MA Siu LY Li M Middle east respiratory syndrome (MERS) coronavirus seroprevalence in domestic livestock in Saudi Arabia, 2010 to 2013. Eurosurveillance (2013) 18(50):21–7. 10.2807/1560-7917.ES2013.18.50.20659 24342517

[24] Reusken CB Ababneh M Raj VS Meyer B Eljarah A Abutarbush S Middle East Respiratory Syndrome coronavirus (MERS-CoV) serology in major livestock species in an affected region in Jordan, June to September 2013. Eurosurveillance (2013) 18(50):20662. 10.2807/1560-7917.ES2013.18.50.20662 24342516

[25] Reusken CB Chrispijn S Raj VS De Bruin E Kohl R Elmoubasher F MERS-CoV infection of Alpaca in a region where MERS-CoV is endemic [letter]. Emerg Infect Dis J (2016) 22(6):1129–31. 10.3201/eid2206.152113 PMC4880085 27070501

[26] Crameri G Durr PA Barr J Yu M Graham K Williams OJ Absence of MERS-CoV antibodies in feral camels in Australia: implications for the pathogen’s origin and spread. One Health (2015) 1:76–82. 10.1016/j.onehlt.2015.10.003 PMC5441328 28616468

[27] Ithete NL Stoffberg S Corman VM Cottontail VM Richards LR Schoeman MC Close relative of human Middle East Respiratory Syndrome coronavirus in bat, South Africa. Emerg Infect Dis (2013) 19(10):1697–9. 10.3201/eid1910.130946 24050621 PMC3810765

[28] Corman VM Ithete NL Richards LR Schoeman MC Preiser W Drosten C Rooting the phylogenetic tree of Middle East Respiratory Syndrome Coronavirus by characterization of a conspecific virus from an African bat. J Virol (2014) 88(19):11297–303. 10.1128/jvi.01498-14 25031349 PMC4178802

[29] Memish ZA Mishra N Olival KJ Fagbo SF Kapoor V Epstein JH Middle East Respiratory Syndrome coronavirus in bats, Saudi Arabia. Emerg Infect Dis (2013) 19(11):1819–23. 10.3201/eid1911.131172 24206838 PMC3837665

[30] Shehata MM Chu DKW Gomaa MR AbiSaid M El Shesheny R Kandeil A Surveillance for coronaviruses in bats, Lebanon and Egypt, 2013–2015. Emerg Infect Dis J (2016) 22(1):148 10.3201/eid2201.151397 PMC4696718 26689887

[31] Alraddadi BM Watson JT Almarashi A Abedi GR Turkistani A Sadran M Risk factors for primary Middle East respiratory syndrome Coronavirus illness in humans, Saudi Arabia, 2014. Emerg Infect Dis (2016) 22(1):49–55. 10.3201/eid2201.151340 26692185 PMC4696714

[32] Müller MA Meyer B Corman VM Al-Masri M Turkestani A Ritz D Presence of Middle East respiratory syndrome coronavirus antibodies in Saudi Arabia: a nationwide, cross-sectional, serological study. Lancet Infect Dis (2015) 15(5):559–64. 10.1016/s1473-3099(15)70090-3 25863564 PMC7185864

[33] Reusken CB Farag EA Haagmans BL Mohran KA Godeke GJ Raj S Occupational exposure to dromedaries and risk for MERS-CoV infection, Qatar, 2013-2014. Emerg Infect Dis J (2015) 21(8):1422–5. 10.3201/eid2108.150481 26196891 PMC4517733

[34] Saudi Arabia Ministry of Health. Media Statements – Corona [Press release]. (2013). Available from: http://www.moh.gov.sa/en/HealthAwareness/Corona/Pages/PressStatements.aspx

[35] Chu DK Oladipo JO Perera RA Kuranga SA Chan SM Poon LL Middle east respiratory syndrome coronavirus (MERS-CoV) in dromedary camels in Nigeria, 2015. Eurosurveillance (2015) 20(49):30086. 10.2807/1560-7917.ES.2015.20.49.30086 26676406

[36] Zyoud SH. Global research trends of Middle East respiratory syndrome coronavirus: a bibliometric analysis. BMC Infect Dis (2016) 16:255. 10.1186/s12879-016-1600-5 27267256 PMC4897912

[37] Goutard FL Paul M Tavornpanich S Houisse I Chanachai K Thanapontharm W Optimizing early detection of avian influenza H5N1 in backyard free-range poultry production systems in Thailand. Prev Vet Med (2012) 105(3):223–34. 10.1016/j.prevetmed.2011.12.020 22296731

[38] Horst HS Dijkhuizen AA Huirne RBM De Leeuw PW Introduction of contagious animal diseases into the Netherlands: elicitation of expert opinions. Livest Prod Sci (1998) 53(3):253–64. 10.1016/S0301-6226(97)00098-5

[39] Shapiro M London B Nigri D Shoss A Zilber E Fogel I Middle East respiratory syndrome coronavirus: review of the current situation in the world. Disast Military Med (2016) 2:9 10.1186/s40696-016-0019-2 PMC5329956 28265443

[40] FAO. Understanding MERS-CoV at the Animal-Human Interface. Summary Report of the Technical Meeting – Rome, Italy. (2016). Available from: http://www.fao.org/documents/card/en/c/a4ed1373-1a0a-4429-8bb8-439c7881c65c/

[41] Saaty RW The analytic hierarchy process – what it is and how it is used. Math Model (1987) 9(3):161–76. 10.1016/0270-0255(87)90473-8

[42] Saaty TL The Analytic Hierarchy Process: Planning, Priority Setting, Resources Allocation. New York, NY: McGraw-Hill (1980). 287 p.

[43] Wind Y Saaty TL Marketing applications of the analytic hierarchy process. Manage Sci (1980) 26(7):641–58. 10.1287/mnsc.26.7.641

[44] Horst HS Huirne RBM Dijkhuizen AA Eliciting the relative importance of risk factors concerning contagious animal diseases using conjoint analysis: a preliminary survey report. Prev Vet Med (1996) 27(3):183–95. 10.1016/0167-5877(95)01003-3

[45] Golden BL Wang Q An alternate measure of consistency. In: Golden BL Wasil EA Harker PT, editors. The Analytic Hierarchy Process: Applications and Studies. Berlin, Heidelberg: Springer (1989). p. 68–81.

[46] Peláez JI Lamata MT A new measure of consistency for positive reciprocal matrices. Comput Math Appl (2003) 46(12):1839–45. 10.1016/S0898-1221(03)90240-9

[47] Liljander A Meyer B Jores J Müller MA Lattwein E Njeru I MERS-CoV antibodies in humans, Africa, 2013–2014. Emerg Infect Dis (2016) 22(6):1086–9. 10.3201/eid2206.160064 27071076 PMC4880087

[48] FAO. Doha Declaration: Regional Workshop on MERS-CoV and One Health. (2015). Available from: http://www.fao.org/ag/againfo/programmes/en/empres/documents/docs/Doha_Declaration_2015.pdf

[49] Hemida MG Chu DKW Poon LLM Perera R Alhammadi MA Ng HY MERS coronavirus in dromedary camel herd, Saudi Arabia. Emerg Infect Dis (2014) 20(7):1231–4. 10.3201/eid2007.140571 24964193 PMC4073860

[50] Reusken CB Farag EA Jonges M Godeke GJ El-Sayed AM Pas SD Middle East respiratory syndrome coronavirus (MERS-CoV) RNA and neutralising antibodies in milk collected according to local customs from dromedary camels, Qatar, April 2014. Eurosurveillance (2014) 19(23):8–12. 10.2807/1560-7917.ES2014.19.23.20829 24957745

[51] van Doremalen N Bushmaker T Munster VJ. Stability of Middle East respiratory syndrome coronavirus (MERS-CoV) under different environmental conditions. Eurosurveillance (2013) 18(38):20590. 10.2807/1560-7917.ES2013.18.38.20590 24084338

[52] Khalafalla AI Lu X Al-Mubarak AIA Dalab AHS Al-Busadah KAS Erdman DD MERS-CoV in upper respiratory tract and lungs of dromedary camels, Saudi Arabia, 2013–2014. Emerg Infect Dis (2015) 21(7):1153 10.3201/eid2107.150070 26079346 PMC4480395

[53] Faye B Madani H El-Rouili S Camel milk value chain in Northern Saudi Arabia. Emir J Food Agric (2014) 26(4):359–65. 10.9755/ejfa.v26i4.17278

[54] WHO. Management of Asymptomatic Persons Who Are RT-PCR Positive for Middle East Respiratory Syndrome Coronavirus (MERS-CoV). (2015). Available from: http://apps.who.int/iris/bitstream/10665/180973/1/WHO_MERS_IPC_15.2_eng.pdf?ua=1&ua=1

[55] WHO. Surveillance for Human Infection with Middle East Respiratory Syndrome Coronavirus (MERS-CoV) Interim Guidance. (2015). Available from: http://apps.who.int/iris/bitstream/10665/177869/1/WHO_MERS_SUR_15.1_eng.pdf?ua=1

[56] Al-Gethamy M Corman VM Hussain R Al-Tawfiq JA Drosten C Memish ZA A case of long-term excretion and subclinical infection with MERS-Coronavirus in a health care worker. Clin Infect Dis (2014) 60(6):973–4. 10.1093/cid/ciu1135 25516193 PMC7108063

[57] Woyessa AB Omballa V Wang D Lambert A Waiboci L Ayele W An outbreak of acute febrile illness caused by Sandfly Fever Sicilian Virus in the Afar region of Ethiopia, 2011. Am J Trop Med Hygiene (2014) 91(6):1250–3. 10.4269/ajtmh.14-0299 25266349 PMC4257654

[58] Lasserre V Guihenneuc-Jouyaux C Richardson S. Biases in ecological studies: utility of including within-area distribution of confounders. Stat Med (2000) 19(1):45–59. 10.1002/(SICI)1097-0258(20000115)19:1<45:AID-SIM276>3.0.CO;2-5 10623912

[59] Wako DD Younan M Tessema TS Glücks IV Baumann MPO. Indigenous knowledge of pastoralists on respiratory diseases of camels in northern Kenya. Prev Vet Med (2016) 130:60–6. 10.1016/j.prevetmed.2016.05.008 27435647

[60] Lawrence MJ Edmundson RH O’Connor MJ The accuracy of combining judgemental and statistical forecasts. Manage Sci (1986) 32(12):1521–32. 10.1287/mnsc.32.12.1521

[61] Seaver DA Assessing Probability with Multiple Individuals: Group Interaction versus Mathematical Aggregation. (1978).

[62] Plous S The Psychology of Judgment and Decision Making. New York, NY: Mcgraw-Hill Book Company (1993).

[63] Funk S Bansal S Bauch CT Eames KTD Edmunds WJ Galvani AP Nine challenges in incorporating the dynamics of behaviour in infectious diseases models. Epidemics (2015) 10:21–5. 10.1016/j.epidem.2014.09.005 25843377

[64] Bradhurst RA Roche SE East IJ Kwan P Garner MG Improving the computational efficiency of an agent-based spatiotemporal model of livestock disease spread and control. Environ Model Softw (2016) 77:1–12. 10.1016/j.envsoft.2015.11.015

[65] Amouroux E Desvaux S Drogoul A Towards virtual epidemiology: an agent-based approach to the modeling of H5N1 propagation and persistence in North-Vietnam. In: Bui TD Ho TV Ha QT, editors. Intelligent Agents and Multi-Agent Systems: 11th Pacific Rim International Conference on Multi-Agents, PRIMA 2008, Hanoi, Vietnam, December 15–16, 2008. Proceedings Berlin, Heidelberg: Springer (2008). p. 26–33.

[66] Macal CM Everything you need to know about agent-based modelling and simulation. J Simul (2016) 10(2):144–56. 10.1057/jos.2016.7

[67] Adney DR van Doremalen N Brown VR Bushmaker T Scott D de Wit E Replication and shedding of MERS-CoV in upper respiratory tract of inoculated dromedary camels. Emerg Infect Dis (2014) 20(12):1999–2005. 10.3201/eid2012.141280 25418529 PMC4257817

[68] Drosten C Seilmaier M Corman VM Hartmann W Scheible G Sack S Clinical features and virological analysis of a case of Middle East respiratory syndrome coronavirus infection. Lancet Infect Dis (2013) 13(9):745–51. 10.1016/s1473-3099(13)70154-3 23782859 PMC7164791

[69] Munster VJ Adney DR van Doremalen N Brown VR Miazgowicz KL Milne-Price S Replication and shedding of MERS-CoV in Jamaican fruit bats (*Artibeus jamaicensis*). Sci Rep (2016) 6:21878. 10.1038/srep21878 26899616 PMC4761889

[70] Corman VM Jores J Meyer B Younan M Liljander A Said MY Antibodies against MERS coronavirus in dromedary camels, Kenya, 1992-2013. Emerg Infect Dis (2014) 20(8):1319–22. 10.3201/eid2008.140596 25075637 PMC4111164

[71] Al-Tawfiq JA Memish ZA. Middle East respiratory syndrome coronavirus: transmission and phylogenetic evolution. Trends Microbiol (2014) 22(10):573–9. 25178651 10.1016/j.tim.2014.08.001 PMC7133228

