# Supplementary material for: MERS-CoV at the Animal–Human Interface: Inputs on Exposure Pathways from an Expert-Opinion Elicitation
Source: Front Vet Sci. 2016 Oct 5;3:88. doi: 10.3389/fvets.2016.00088 (PMC5051548; doi:10.3389/fvets.2016.00088)
Supplement: Supplementary file 1 [file image_1.PDF]

MERS Transmission Model: Expert Opinion Elicitation

Background Information

Dear expert,

Welcome to the online questionnaire! Thank you for your willingness to participate in our research.

The purpose of this questionnaire is to extract your estimations on the general probabilities of certain types of MERS transmission, relative importance of risk factors, and your perceived level of confidence for each of your answers. The information you give us will be used to fill in gaps in a MERS-CoV transmission model; specifically, to inform us about highly likely and unlikely pathways and highlight areas that deserve increased attention for field surveys and studies.

For example, please see the sample model below which highlights possible transmission pathways and research questions which have been suggested and reported on in the literature, up until now.

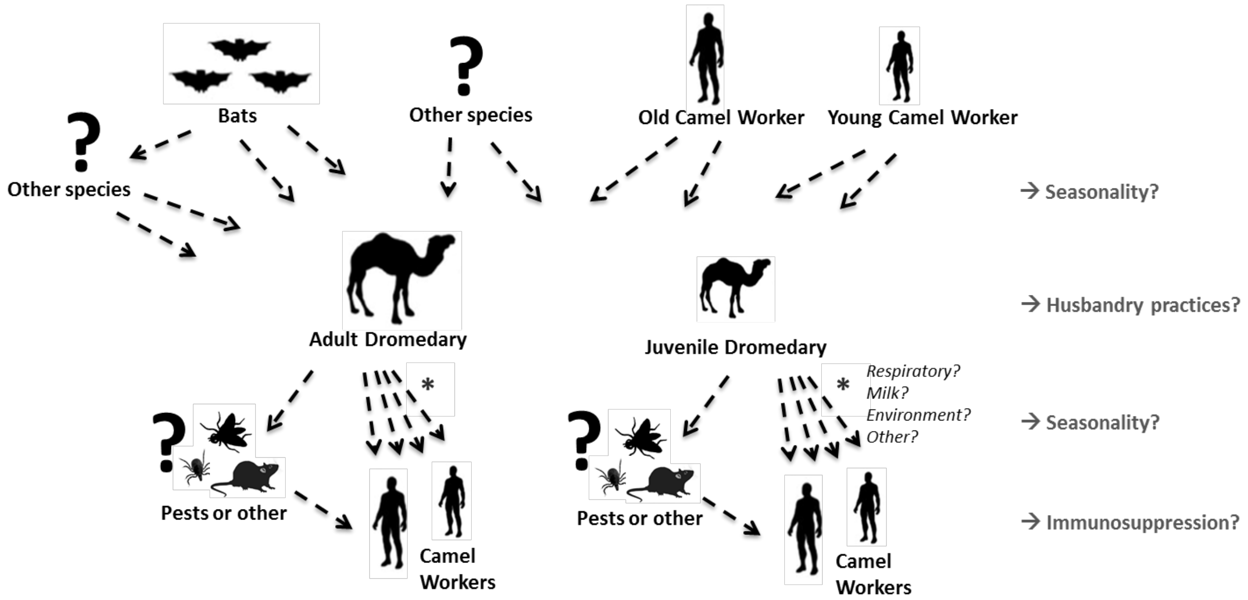

The point of this questionnaire is to obtain:

- your expert opinion on the plausibility and importance of some of the hypothesized, but not yet evidenced, pathways
- your estimation of the transmission risks from some of the evidenced, but not yet solidly quantified, pathways
- your feedback about any other transmission pathways or important factors that have been missed

MERS Transmission Model: Expert Opinion Elicitation

Questionnaire Instructions

General instructions and points to remember for filling out the survey:

*Please pay attention to specifications in the questions given (ex. age of camel worker, age of dromedary, etc). These items are **bolded**, put in *italics*, or underlined as much as possible

*You can scroll through the questionnaire by pressing the “Next” and “Back” buttons. Though to ensure you do not lose your answers on a page: press ‘next’ before going back!

*By pressing “Finish” on the last page you will finalise the questionnaire, which will be then sent to us automatically.

*Finally, **please answer all questions**, even if you are not 100% confident in your answer. The purpose of this exercise is to obtain your 'best guess' to questions that have not yet been solidly quantified, so uncertainty is understandable. You will be given the chance to state your level of confidence for each of your answers, and this will be taken into consideration in the final model.

The questionnaire consists of 5 pair-wise comparison of risk factor questions, 3 scenario-based transmission probability questions, and 3 open ended questions. You will also be asked for a few details about your background at the beginning of the questionnaire, and for any final comments (etc) that you may have at the end. As mentioned in the invitation email, we estimate that it will take you approximately 40 minutes to complete. **Please note that all your answers and comments will be anonymised.**

If you have any difficulties or questions, please contact Anna Funk at anna-louise.funk@pasteur.fr

MERS Transmission Model: Expert Opinion Elicitation

Expert Background

* What is your first and last name?

Please give your general background

- Non medical diploma
- Medical doctor (MD)
- Doctor of veterinary medicine (DVM)
- Other (please specify)

Please give details on your expertise

	Here, options are: "Yes", "No"<br>Expertise	Here, options are: 1-5, 6-10, 10+ years<br>Number of Years Experience
Epidemiology		
Virology		
Camel Research/Camel clinical Studies		
Risk Analysis or Modeling		
MERS-CoV virology		
MERS-CoV epidemiology		
Chiropterology (study of bats)		

EXAMPLE: Pair-Wise Comparison of Risk Factors

Training and Example on Filling Out Pair-Wise Comparison Questions

Please read carefully

In each of the following questions in this section you will be FIRST be asked which risk factors you think are associated with each infection scenario.

Lets take the topic of malaria as an example, the question and your answer will look like what is seen below. This is if, lets pretend, you had selected those items as possible risk factors:

Identify the risk factors associated with a high malaria incidence

Check the boxes corresponding to risk factors you believe associated with malaria incidence.

- [x] Density of anopheles mosquitoes
- [x] Proximity to open water areas
- [ ] Proportion of people suffering from malnutrition
- [x] Typical housing structure is open/unenclosed
- [ ] Other (please specify)

You will THEN be asked to describe the relationship that each selected risk factor has which each other:

Again, with the example of malaria incidence, and its relationship with the risk factor "density of Anopheles mosquitoes". When comparing this risk factor to others, 9 different comparative options are possible, ranging from "extremely more important" (weight of 9) to "extremely less important" (weight of 1/9).

In this example: if the risk factor "density of Anopheles mosquitoes" is considered to be very strongly less important than the risk factor "proximity to open water area" for the risk of malaria, then check the box as below.

In the same way, if the risk factor "density of Anopheles mosquitoes" is considered to be very strongly more important than the risk factor "proportion of people suffering from malnutrition", then check the box as below.

More important								Less important
Extremely	Very strongly	Strongly	Moderately	**Equivalent**	Moderately	Strongly	Very strongly	Extremely
9	7	5	3	1	1/3	1/5	1/7	1/9

Density of Anopheles mosquitoes is

	9: extremely more important than	7: very strongly more important than	5: strongly more important than	3: moderately more important than	1: equivalent to	1/3: moderately less important than	1/5: strongly less important than	1/7: very strongly less important than	1/9: extremely less important than
Proximity to open water area	○	○	○	○	○	○	○	✓	○
Proportion of people suffering from malnutrition	○	✓	○	○	○	○	○	○	○

NOTE, in the pair-wise comparison question, tables will be given for ALL risk factors in the original list. However, **you do not need to fill out any table or any row for a risk factor which you did not select** in Step 1 of that question.

--> If the title/heading risk factor for the table is not one that you have selected in Step 1, please skip that table and move to the next.

--> If one of the tables contains a risk factor which you did not select in Step 1, do not tick any boxes in that row, but do fill out all other rows in the table.

If we continue with the malaria example, and the risk factors selected above, your entire answer could look like this:

Density of anopheles mosquitoes is...

		9: extremely more important than	7: very strongly more important than	5: strongly more important than	3: moderately more important than	1: equal to	1/3: moderately less important than	1/5: strongly less important than	1/7: very strongly less important than	1/9: extremely less important than
✔	Proximity to open water areas	○	○	○	●	○	○	○	○	○
✖	Proportion of people suffering from malnutrition	○	-----------------	-----------------	-----------------	-Do not fill out-	-----------------	-----------------	○	○
✔	Typical housing structure is open/unenclosed	○	○	○	○	●	○	○	○	○
✖	Other (*if you've specified an 'other' in Step 1*)	○	-----------------	-----------------	-----------------	-Do not fill out-	-----------------	-----------------	○	○

If you have included 'other', please specify

Proximity to open water areas is...

		9: extremely more important than	7: very strongly more important than	5: strongly more important than	3: moderately more important than	1: equal to	1/3: moderately less important than	1/5: strongly less important than	1/7: very strongly less important than	1/9: extremely less important than
✖	Proportion of people suffering from malnutrition	○	-----------------	-----------------	-----------------	-Do not fill out-	-----------------	-----------------	○	○
✔	Typical housing structure is open/unenclosed	○	○	○	○	○	●	○	○	○
✖	Other (*if you've specified an 'other' in Step 1*)	○	-----------------	-----------------	-----------------	-Do not fill out-	-----------------	-----------------	○	○

If you have included 'other', please specify

Proportion of people suffering from malnutrition is... Skip entire table as did not select this risk factor

	**9:** extremely more important than	**7:** very strongly more important than	**5:** strongly more important than	**3:** moderately more important than	**1:** equal to	[illegible]	**1/5:** strongly less important than	**1/7:** very strongly less important than	**1/9:** extremely less important than
Typical housing structure is open/unenclosed	○	○	○	○	○	○	○	○	○
Other (*if you've specified an 'other' in Step 1*)	○	○	○	○	○	○	○	○	○

If you have included 'other', please specify

Typical housing structure is open/unenclosed is...

	**9:** extremely more important than	**7:** very strongly more important than	**5:** strongly more important than	**3:** moderately more important than	**1:** equal to	**1/3:** moderately less important than	**1/5:** strongly less important than	**1/7:** very strongly less important than	**1/9:** extremely less important than
Other (*if you've specified an 'other' in Step 1*)	○	○	○	○	○	○	○	○	○

-------------------Do not fill out---------------------------

If you have included 'other', please specify

Question 1

Step 1: Identify the intrinsic and extrinsic risk factors for a dromedary camel (from a currently un-infected herd) to become infected with MERS-CoV

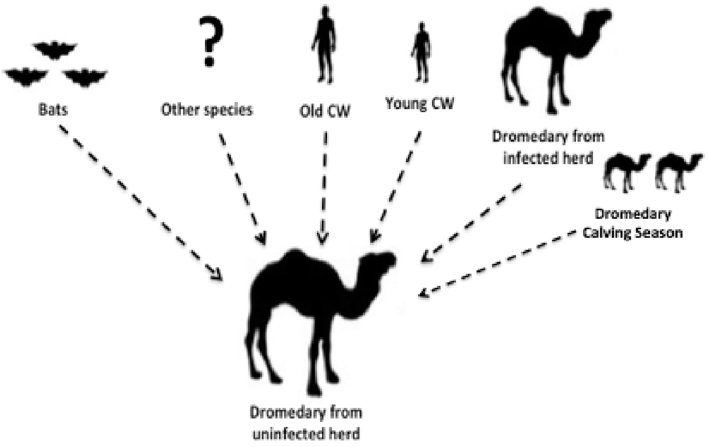

Check the boxes corresponding to risk factors you believe associated with camel infection.

- [ ] Infestation of MERS-CoV infected bats in close proximity
- [ ] Daily, close contact with a MERS-CoV infected camel worker (*greater than 50 years old*)
- [ ] Daily, close contact with a MERS-CoV infected camel worker (*less than or equal to 50 years old*)
- [ ] Short term contact/crossing with another herd of dromedaries which is MERS-CoV infected
- [ ] Short term contact/crossing with another species (not dromedaries) which is MERS-CoV infected
- [ ] Case's occurence coincides with dromedary calving season (ex. December to February in the Arabian Peninsula)
- [ ] Other (please specify)

Please enter your confidence level for your selection of risk factors in the previous question

	**1:** Completely unsure	**2:** Confident for a small part of answer only	**3:** Fairly confident; multiple doubts	**4:** Confident, with a few minor doubts	**5**: Very confident
Confidence level	◯	◯	◯	◯	◯

Question 1

Step 2: Select an option according to the influence that each risk factor has on a dromedary (from an uninfected herd) becoming infected with MERS-CoV

IMPORTANT NOTICE: In each of the tables below, you only need to provide answers for the risk factors that you have selected in Step 1 of this question.

If the title/heading risk factor for the table is not one that you have selected in Step 1, please skip that table and move to the next. If one of the tables contains a risk factor which you did not select in Step 1, do not tick any boxes in that row, but DO fill out all other rows in the table.

Infestation of MERS-CoV infected bats in close proximity is...

	**9:** extremely more important than	**7:** very strongly more important than	**5:** strongly more important than	**3:** moderately more important than	**1:** equal to	**1/3:** moderately less important than	**1/5:** strongly less important than	**1/7:** very strongly less important than	**1/9:** extremely less important than
Daily, close contact with infected camel worker (*greater than 50 years old*)	◯	◯	◯	◯	◯	◯	◯	◯	◯
Daily, close contact with infected camel worker (*less than or equal to 50 years old*)	◯	◯	◯	◯	◯	◯	◯	◯	◯
Short term contact/crossing with another dromedary herd that is MERS-CoV infected	◯	◯	◯	◯	◯	◯	◯	◯	◯
Short term contact/crossing with other species (not dromedaries) that is MERS-CoV infected	◯	◯	◯	◯	◯	◯	◯	◯	◯
Case's occurrence coincides with dromedary calving season (ex. December to February in the Arabian Peninsula)	◯	◯	◯	◯	◯	◯	◯	◯	◯
Other (*if you've specified an 'other' in Step 1*)	◯	◯	◯	◯	◯	◯	◯	◯	◯

If you have included 'other', please specify

Daily, close contact with infected camel worker (greater than 50 years old) is...

	**9:** extremely more important than	**7:** very strongly more important than	**5:** strongly more important than	**3:** moderately more important than	**1:** equal to	**1/3:** moderately less important than	**1/5:** strongly less important than	**1/7:** very strongly less important than	**1/9:** extremely less important than
Daily, close contact with infected camel worker (*less than or equal to 50 years old*)	○	○	○	○	○	○	○	○	○
Short term contact/crossing with another dromedary herd that is MERS-CoV infected	○	○	○	○	○	○	○	○	○
Short term contact/crossing with other species (not dromedaries) that is MERS-CoV infected	○	○	○	○	○	○	○	○	○
Case's occurrence coincides with dromedary calving season (ex. December to February in the Arabian Peninsula)	○	○	○	○	○	○	○	○	○
Other (*if you've specified an 'other' in Step 1*)	○	○	○	○	○	○	○	○	○

If you have included 'other', please specify

Daily, close contact with infected camel worker (less than or equal to 50 years old) is...

	**9:** extremely more important than	**7:** very strongly more important than	**5:** strongly more important than	**3:** moderately more important than	**1:** equal to	**1/3:** moderately less important than	**1/5:** strongly less important than	**1/7:** very strongly less important than	**1/9:** extremely less important than
Short term contact/crossing with another dromedary herd that is MERS-CoV infected	◯	◯	◯	◯	◯	◯	◯	◯	◯
Short term contact/crossing with other species (not dromedaries) that is MERS-CoV infected	◯	◯	◯	◯	◯	◯	◯	◯	◯
Case's occurrence coincides with dromedary calving season (ex. December to February in the Arabian Peninsula)	◯	◯	◯	◯	◯	◯	◯	◯	◯
Other (*if you've specified an 'other' in Step 1*)	◯	◯	◯	◯	◯	◯	◯	◯	◯

If you have included 'other', please specify

Short term contact/crossing with another dromedary herd that is MERS-CoV infected is...

	**9:** extremely more important than	**7:** very strongly more important than	**5:** strongly more important than	**3:** moderately more important than	**1:** equal to	**1/3:** moderately less important than	**1/5:** strongly less important than	**1/7:** very strongly less important than	**1/9:** extremely less important than
Short term contact/crossing with other species (not dromedaries) that is MERS-CoV infected	○	○	○	○	○	○	○	○	○
Case's occurrence coincides with dromedary calving season (ex. December to February in the Arabian Peninsula)	○	○	○	○	○	○	○	○	○
Other (*if you've specified an 'other' in Step 1*)	○	○	○	○	○	○	○	○	○

If you have included 'other', please specify

Short term contact/crossing with other species (not dromedaries) that is MERS-CoV infected is...

	**9:** extremely more important than	**7:** very strongly more important than	**5:** strongly more important than	**3:** moderately more important than	**1:** equal to	**1/3:** moderately less important than	**1/5:** strongly less important than	**1/7:** very strongly less important than	**1/9:** extremely less important than
Case's occurrence coincides with dromedary calving season (ex. December to February in the Arabian Peninsula)	○	○	○	○	○	○	○	○	○
Other (*if you've specified an 'other' in Step 1*)	○	○	○	○	○	○	○	○	○

If you have included 'other', please specify

Season coincides with dromedary calving season (ex. December to February in the Arabian Peninsula) is...

	**9:** extremely more important than	**7:** very strongly more important than	**5:** strongly more important than	**3:** moderately more important than	**1:** equal to	**1/3:** moderately less important than	**1/5:** strongly less important than	**1/7:** very strongly less important than	**1/9:** extremely less important than
Other (*if you've specified an 'other' in Step 1*)	○	○	○	○	○	○	○	○	○

If you have included 'other', please specify

Risk Factors and Pair-Wise Comparisons: Question 1

Please enter your general confidence level for the pairwise comparisons you did on the last page for Question 1, Step 2

	**1:** Completely unsure	**2:** Confident for a small part of answer only	**3:** Fairly confident; multiple doubts	**4:** Confident, with a few minor doubts	**5**: Very confident
Confidence level	○	○	○	○	○

Question 2

Step 1: Identify the risk factors for an uninfected dromedary herd to be infected by another herd.

Check the boxes corresponding to risk factors you believe associated with herd infection.

- [ ] The uninfected dromedary herd is nomadic
- [ ] There is introduction of a new dromedary into the uninfected herd (ex. bought, loaned, special occasion, breeding purposes)
- [ ] The uninfected herd inhabits an area which is high in dromedary camel density
- [ ] The uninfected herd is taken to racetracks
- [ ] The uninfected herd is taken to beauty contests
- [ ] The uninfected herd is taken to communal waterpoints
- [ ] The uninfected herd is taken to markets
- [ ] The uninfected herd is taken through border areas
- [ ] Other (please specify)

Please enter your confidence level for your selection of risk factors in the previous question

	**1:** Completely unsure	**2:** Confident for a small part of answer only	**3:** Fairly confident; multiple doubts	**4:** Confident, with a few minor doubts	**5**: Very confident
Confidence level	◯	◯	◯	◯	◯

Question 2

Step 2: Select an option according to the influence that each risk factor has on an uninfected dromedary herd being infected by another herd

IMPORTANT NOTICE: In each of the tables below, you only need to provide answers for the risk factors that you have selected in Step 1 of this question.

If the title/heading risk factor for the table is not one that you have selected in Step 1, please skip that table and move to the next. If one of the tables contains a risk factor which you did not select in Step 1, do not tick any boxes in that row, but DO fill out all other rows in the table.

The uninfected dromedary herd being nomadic is...

	**9:** extremely more important than	**7:** very strongly more important than	**5:** strongly more important than	**3:** moderately more important than	**1:** equal to	**1/3:** moderately less important than	**1/5:** strongly less important than	**1/7:** very strongly less important than	**1/9:** extremely less important than
There is introduction of a new dromedary into the herd (ex. bought, loaned, special occasion, breeding purposes)									
The uninfected herd inhabits an area which is high in dromedary camel density									
The uninfected herd is taken to race tracks									
The uninfected herd is taken to beauty contests									
The uninfected herd is taken to communal water-points									
The uninfected herd is taken to markets									
The uninfected herd is taken through border areas									
Other (*if you've specified an 'other' in Step 1*)									

If you have included 'other', please specify

Introduction of a new dromedary into the herd (ex. bought, loaned, special occasion, breeding purposes) is...

	**9:** extremely more important than	**7:** very strongly more important than	**5:** strongly more important than	**3:** moderately more important than	**1:** equal to	**1/3:** moderately less important than	**1/5:** strongly less important than	**1/7:** very strongly less important than	**1/9:** extremely less important than
The uninfected herd inhabits an area which is high in dromedary camel density	○	○	○	○	○	○	○	○	○
The uninfected herd is taken to race tracks	○	○	○	○	○	○	○	○	○
The uninfected herd is taken to beauty contests	○	○	○	○	○	○	○	○	○
The uninfected herd is taken to communal water-points	○	○	○	○	○	○	○	○	○
The uninfected herd is taken to markets	○	○	○	○	○	○	○	○	○
The uninfected herd is taken through border areas	○	○	○	○	○	○	○	○	○
Other (*if you've specified an 'other' in Step 1*)	○	○	○	○	○	○	○	○	○

If you have included 'other', please specify

The uninfected herd inhabiting an area which is high in dromedary camel density is...

	**9:** extremely more important than	**7:** very strongly more important than	**5:** strongly more important than	**3:** moderately more important than	**1:** equal to	**1/3:** moderately less important than	**1/5:** strongly less important than	**1/7:** very strongly less important than	**1/9:** extremely less important than
The uninfected herd is taken to race tracks									
The uninfected herd is taken to beauty contests									
The uninfected herd is taken to communal water-points									
The uninfected herd is taken to markets									
The uninfected herd is taken through border areas									
Other (*if you've specified an 'other' in Step 1*)									

If you have included 'other', please specify

The uninfected herd being taken to race tracks is...

	**9:** extremely more important than	**7:** very strongly more important than	**5:** strongly more important than	**3:** moderately more important than	**1:** equal to	**1/3:** moderately less important than	**1/5:** strongly less important than	**1/7:** very strongly less important than	**1/9:** extremely less important than
The uninfected herd is taken to beauty contests									
The uninfected herd is taken to communal water-points									
The uninfected herd is taken to markets									
The uninfected herd is taken through border areas									
Other (*if you've specified an 'other' in Step 1*)									

If you have included 'other', please specify

The uninfected herd being taken to beauty contests is...

	**9:** extremely more important than	**7:** very strongly more important than	**5:** strongly more important than	**3:** moderately more important than	**1:** equal to	**1/3:** moderately less important than	**1/5:** strongly less important than	**1/7:** very strongly less important than	**1/9:** extremely less important than
The uninfected herd is taken to communal water-points	○	○	○	○	○	○	○	○	○
The uninfected herd is taken to markets	○	○	○	○	○	○	○	○	○
The uninfected herd is taken through border areas	○	○	○	○	○	○	○	○	○
Other (*if you've specified an 'other' in Step 1*)	○	○	○	○	○	○	○	○	○

If you have included 'other', please specify

The uninfected herd being taken to communal water-points is...

	**9:** extremely more important than	**7:** very strongly more important than	**5:** strongly more important than	**3:** moderately more important than	**1:** equal to	**1/3:** moderately less important than	**1/5:** strongly less important than	**1/7:** very strongly less important than	**1/9:** extremely less important than
The uninfected herd is taken to markets	○	○	○	○	○	○	○	○	○
The uninfected herd is taken through border areas	○	○	○	○	○	○	○	○	○
Other (*if you've specified an 'other' in Step 1*)	○	○	○	○	○	○	○	○	○

If you have included 'other', please specify

The uninfected herd being taken to markets is...

	**9:** extremely more important than	**7:** very strongly more important than	**5:** strongly more important than	**3:** moderately more important than	**1:** equal to	**1/3:** moderately less important than	**1/5:** strongly less important than	**1/7:** very strongly less important than	**1/9:** extremely less important than
The uninfected herd is taken through border areas	○	○	○	○	○	○	○	○	○
Other (*if you've specified an 'other' in Step 1*)	○	○	○	○	○	○	○	○	○

If you have included 'other', please specify

The uninfected herd being taken through border areas is...

	**9:** extremely more important than	**7:** very strongly more important than	**5:** strongly more important than	**3:** moderately more important than	**1:** equal to	**1/3:** moderately less important than	**1/5:** strongly less important than	**1/7:** very strongly less important than	**1/9:** extremely less important than
Other (*if you've specified an 'other' in Step 1*)	○	○	○	○	○	○	○	○	○

If you have included 'other', please specify

MERS Transmission Model: Expert Opinion Elicitation

Risk Factors and Pair-Wise Comparisons: Question 2

Please enter your general confidence level for the pairwise comparisons you did on the last page for Question 2, Step 2

	**1:** Completely unsure	**2:** Confident for a small part of answer only	**3:** Fairly confident; multiple doubts	**4:** Confident, with a few minor doubts	**5**: Very confident
Confidence level	○	○	○	○	○

Question 3

Step 1: Identify the risk factors for occurrence of a human primary case of MERS-CoV

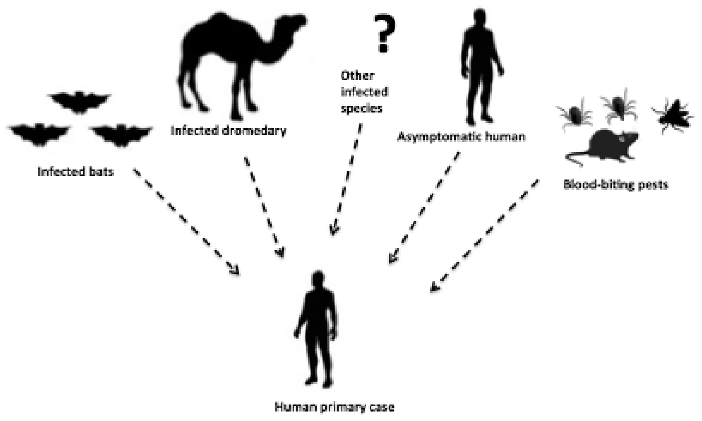

Check the boxes corresponding to risk factors you believe associated with human infection.

- ☐ There is an infestation of MERS-CoV infected bats in close proximity
- ☐ The human has contact with an infected herd of dromedary camels
- ☐ The human has contact with another animal species (not dromedary camels) that is MERS-CoV infected
- ☐ Blood biting pests (ex. fleas, ticks) are infesting a MERS-CoV infected animal species as well as the human
- ☐ The human has contact with another human who is MERS-CoV infected but asymptomatic
- ☐ Other (please specify)

Please enter your confidence level for your selection of risk factors in the previous question

	**1:** Completely unsure	**2:** Confident for a small part of answer only	**3:** Fairly confident; multiple doubts	**4:** Confident, with a few minor doubts	**5**: Very confident
Confidence level	○	○	○	○	○

Question 3

Step 2: Select an option according to the influence that each risk factor has on occurence of a human primary case of MERS-CoV

IMPORTANT NOTICE: In each of the tables below, you only need to provide answers for the risk factors that you have selected in Step 1 of this question.

If the title/heading risk factor for the table is not one that you have selected in Step 1, please skip that table and move to the next. If one of the tables contains a risk factor which you did not select in Step 1, do not tick any boxes in that row, but DO fill out all other rows in the table.

An infestation of MERS-CoV infected bats in close proximity is...

	**9:** extremely more important than	**7:** very strongly more important than	**5:** strongly more important than	**3:** moderately more important than	**1:** equal to	**1/3:** moderately less important than	**1/5:** strongly less important than	**1/7:** very strongly less important than	**1/9:** extremely less important than
The human has contact with a MERS-CoV infected herd of dromedary camels	○	○	○	○	○	○	○	○	○
The human has contact with another animal species (not dromedary camels) that is MERS-CoV infected	○	○	○	○	○	○	○	○	○
Blood biting pests (ex. fleas, ticks) are infesting a MERS-CoV infected animal species as well as the human	○	○	○	○	○	○	○	○	○
The human has contact with another human who is MERS-CoV infected but asymptomatic	○	○	○	○	○	○	○	○	○
Other (*if you've specified an 'other' in Step 1*)	○	○	○	○	○	○	○	○	○

If you have included 'other', please specify

The human having contact with a MERS-CoV infected herd of dromedary camels is...

	**9:** extremely more important than	**7:** very strongly more important than	**5:** strongly more important than	**3:** moderately more important than	**1:** equal to	**1/3:** moderately less important than	**1/5:** strongly less important than	**1/7:** very strongly less important than	**1/9:** extremely less important than
The human has contact with another animal species (not dromedary camels) that is MERS-CoV infected	○	○	○	○	○	○	○	○	○
Blood biting pests (ex. fleas, ticks) are infesting a MERS-CoV infected animal species as well as the human	○	○	○	○	○	○	○	○	○
The human has contact with another human who is MERS-CoV infected but asymptomatic	○	○	○	○	○	○	○	○	○
Other (*if you've specified an 'other' in Step 1*)	○	○	○	○	○	○	○	○	○

If you have included 'other', please specify

The human having contact with another animal species (not dromedary camels) that is MERS-CoV infected is...

	**9:** extremely more important than	**7:** very strongly more important than	**5:** strongly more important than	**3:** moderately more important than	**1:** equal to	**1/3:** moderately less important than	**1/5:** strongly less important than	**1/7:** very strongly less important than	**1/9:** extremely less important than
Blood biting pests (ex. fleas, ticks) are infesting a MERS-CoV infected animal species as well as the human	○	○	○	○	○	○	○	○	○
The human has contact with another human who is MERS-CoV infected but asymptomatic	○	○	○	○	○	○	○	○	○
Other (*if you've specified an 'other' in Step 1*)	○	○	○	○	○	○	○	○	○

If you have included 'other', please specify

Blood biting pests (ex. fleas, ticks) are infesting a MERS-CoV infected animal species as well as the human is...

	**9:** extremely more important than	**7:** very strongly more important than	**5:** strongly more important than	**3:** moderately more important than	**1:** equal to	**1/3:** moderately less important than	**1/5:** strongly less important than	**1/7:** very strongly less important than	**1/9:** extremely less important than
The human has contact with another human who is MERS-CoV infected but asymptomatic	◯	◯	◯	◯	◯	◯	◯	◯	◯
Other (*if you've specified an 'other' in Step 1*)	◯	◯	◯	◯	◯	◯	◯	◯	◯

If you have included 'other', please specify

The human has contact with another human who is MERS-CoV infected but asymptomatic is...

	**9:** extremely more important than	**7:** very strongly more important than	**5:** strongly more important than	**3:** moderately more important than	**1:** equal to	**1/3:** moderately less important than	**1/5:** strongly less important than	**1/7:** very strongly less important than	**1/9:** extremely less important than
Other (*if you've specified an 'other' in Step 1*)	◯	◯	◯	◯	◯	◯	◯	◯	◯

If you have included 'other', please specify

MERS Transmission Model: Expert Opinion Elicitation

Risk Factors and Pair-Wise Comparisons: Question 3

Please enter your general confidence level for the pairwise comparisons you did on the last page for Question 3, Step 2

	**1:** Completely unsure	**2:** Confident for a small part of answer only	**3:** Fairly confident; multiple doubts	**4:** Confident, with a few minor doubts	**5**: Very confident
Confidence level	○	○	○	○	○

Do you believe that MERS-CoV infected camels can transmit the virus to humans, and/or play a role in humans becoming infected with MERS-CoV?

- ◯ Yes
- ◯ No

Question 4

Step 1: Identify the risk factors for a camel worker to become INFECTED (viraemia positive) by a MERS-CoV infected dromedary

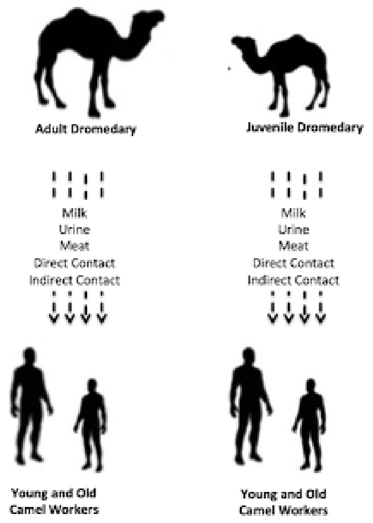

Check the boxes corresponding to risk factors you believe associated with camel worker infection.

☐ Camel workers are drinking unpasteurized milk from the MERS-CoV infected dromedaries

☐ Camel workers are drinking/using urine from the MERS-CoV infected dromedaries

☐ Camel workers are eating raw meat from the MERS-CoV infected dromedaries

☐ Camel workers have direct, close contact (ex. touching, kissing, assisting in births, milking, administering medicines, slaughtering) with infected dromedaries

☐ Camel workers have indirect contact (ex. cleaning animal housing area, handling animal waste) with infected dromedaries

☐ Other (please specify)

Please enter your confidence level for your selection of risk factors in the previous question

	**1:** Completely unsure	**2:** Confident for a small part of answer only	**3:** Fairly confident; multiple doubts	**4:** Confident, with a few minor doubts	**5**: Very confident
Confidence level	○	○	○	○	○

Question 4

Step 2: Select an option according to the influence that each risk factor has on a camel worker becoming INFECTED (viraemia positive) by a MERS-CoV infected dromedary

IMPORTANT NOTICE: In each of the tables below, you only need to provide answers for the risk factors that you have selected in Step 1 of this question.

If the title/heading risk factor for the table is not one that you have selected in Step 1, please skip that table and move to the next. If one of the tables contains a risk factor which you did not select in Step 1, do not tick any boxes in that row, but DO fill out all other rows in the table.

Camel workers drinking unpasteurized milk from the MERS-CoV infected dromedaries is...

	**9:** extremely more important than	**7:** very strongly more important than	**5:** strongly more important than	**3:** moderately more important than	**1:** equal to	**1/3:** moderately less important than	**1/5:** strongly less important than	**1/7:** very strongly less important than	**1/9:** extremely less important than
Camel workers are drinking/using urine from the MERS-CoV infected dromedaries	○	○	○	○	○	○	○	○	○
Camel workers are eating raw meat from the MERS-CoV infected dromedaries	○	○	○	○	○	○	○	○	○
Camel workers have direct, close contact (ex. touching, kissing, assisting in births, milking, administering medicines, slaughtering) with infected dromedaries	○	○	○	○	○	○	○	○	○
Camel workers have indirect contact (ex. cleaning animal housing area, handling animal waste) with infected dromedaries	○	○	○	○	○	○	○	○	○
Other (*if you've specified an 'other' in Step 1*)	○	○	○	○	○	○	○	○	○

If you have included 'other', please specify

Camel workers drinking/using urine from the MERS-CoV infected dromedaries is...

	**9:** extremely more important than	**7:** very strongly more important than	**5:** strongly more important than	**3:** moderately more important than	**1:** equal to	**1/3:** moderately less important than	**1/5:** strongly less important than	**1/7:** very strongly less important than	**1/9:** extremely less important than
Camel workers are eating raw meat from the MERS-CoV infected dromedaries	○	○	○	○	○	○	○	○	○
Camel workers have direct, close contact (ex. touching, kissing, assisting in births, milking, administering medicines, slaughtering) with infected dromedaries	○	○	○	○	○	○	○	○	○
Camel workers have indirect contact (ex. cleaning animal housing area, handling animal waste) with infected dromedaries	○	○	○	○	○	○	○	○	○
Other (*if you've specified an 'other' in Step 1*)	○	○	○	○	○	○	○	○	○

If you have included 'other', please specify

Camel workers eating raw meat from the MERS-CoV infected dromedaries is...

	**9:** extremely more important than	**7:** very strongly more important than	**5:** strongly more important than	**3:** moderately more important than	**1:** equal to	**1/3:** moderately less important than	**1/5:** strongly less important than	**1/7:** very strongly less important than	**1/9:** extremely less important than
Camel workers have direct, close contact (ex. touching, kissing, assisting in births, milking, administering medicines, slaughtering) with infected dromedaries	○	○	○	○	○	○	○	○	○
Camel workers have indirect contact (ex. cleaning animal housing area, handling animal waste) with infected dromedaries	○	○	○	○	○	○	○	○	○
Other (*if you've specified an 'other' in Step 1*)	○	○	○	○	○	○	○	○	○

If you have included 'other', please specify

Camel workers having direct, close contact (ex. touching, kissing, assisting in births, milking, administering medicines, slaughtering) with infected dromedaries is...

	**9:** extremely more important than	**7:** very strongly more important than	**5:** strongly more important than	**3:** moderately more important than	**1:** equal to	**1/3:** moderately less important than	**1/5:** strongly less important than	**1/7:** very strongly less important than	**1/9:** extremely less important than
Camel workers have indirect contact (ex. cleaning animal housing area, handling animal waste) with infected dromedaries	○	○	○	○	○	○	○	○	○
Other (*if you've specified an 'other' in Step 1*)	○	○	○	○	○	○	○	○	○

If you have included 'other', please specify

Camel workers having indirect contact (ex. cleaning animal housing area, handling animal waste) with infected dromedaries is...

	**9:** extremely more important than	**7:** very strongly more important than	**5:** strongly more important than	**3:** moderately more important than	**1:** equal to	**1/3:** moderately less important than	**1/5:** strongly less important than	**1/7:** very strongly less important than	**1/9:** extremely less important than
Other (*if you've specified an 'other' in Step 1*)	○	○	○	○	○	○	○	○	○

If you have included 'other', please specify

MERS Transmission Model: Expert Opinion Elicitation

Risk Factors and Pair-Wise Comparisons: Question 4

Please enter your general confidence level for the pairwise comparisons you did on the last page for Question 4, Step 2

	**1:** Completely unsure	**2:** Confident for a small part of answer only	**3:** Fairly confident; multiple doubts	**4:** Confident, with a few minor doubts	**5**: Very confident
Confidence level	○	○	○	○	○

Question 5

Step 1: Identify the risk factors for a MERS-CoV infected camel worker becoming SYMPTOMATIC/SICK

Check the boxes corresponding to risk factors you believe associated with humans becoming symptomatic/sick.

- ☐ Camel worker is > 50 years of age
- ☐ Camel worker is immunocompromised/has underlying medical condition
- ☐ Camel worker has a genetic disorder
- ☐ Timing of infection coincides with, or directly follows, other epidemic (ex. influenza, other CoVs)
- ☐ Amount of viral dose transmitted
- ☐ Other (please specify)

Please enter your confidence level for your selection of risk factors in the previous question

	**1:** Completely unsure	**2:** Confident for a small part of answer only	**3:** Fairly confident; multiple doubts	**4:** Confident, with a few minor doubts	**5**: Very confident
Confidence level	○	○	○	○	○

Question 5

Step 2: Select an option according to the influence that each risk factor has on a MERS-CoV infected camel worker becoming SYMPTOMATIC/SICK

IMPORTANT NOTICE: In each of the tables below, you only need to provide answers for the risk factors that you have selected in Step 1 of this question.

If the title/heading risk factor for the table is not one that you have selected in Step 1, please skip that table and move to the next. If one of the tables contains a risk factor which you did not select in Step 1, do not tick any boxes in that row, but DO fill out all other rows in the table.

Camel worker being greater than 50 years of age is...

	**9:** extremely more important than	**7:** very strongly more important than	**5:** strongly more important than	**3:** moderately more important than	**1:** equal to	**1/3:** moderately less important than	**1/5:** strongly less important than	**1/7:** very strongly less important than	**1/9:** extremely less important than
Camel worker being immunocompromised/having underlying medical condition	◯	◯	◯	◯	◯	◯	◯	◯	◯
Camel worker having a genetic disorder	◯	◯	◯	◯	◯	◯	◯	◯	◯
Timing of infection coincides with, or directly follows, other epidemic (ex. influenza, other CoVs)	◯	◯	◯	◯	◯	◯	◯	◯	◯
Amount of viral dose transmitted	◯	◯	◯	◯	◯	◯	◯	◯	◯
Other (*if you've specified an 'other' in Step 1*)	◯	◯	◯	◯	◯	◯	◯	◯	◯

If you have included 'other', please specify

Camel worker being immunocompromised/having underlying medical condition is...

	**9:** extremely more important than	**7:** very strongly more important than	**5:** strongly more important than	**3:** moderately more important than	**1:** equal to	**1/3:** moderately less important than	**1/5:** strongly less important than	**1/7:** very strongly less important than	**1/9:** extremely less important than
Camel worker having a genetic disorder									
Timing of infection coincides with, or directly follows, other epidemic (ex. influenza, other CoVs)									
Amount of viral dose transmitted									
Other (*if you've specified an 'other' in Step 1*)									

If you have included 'other', please specify

Camel worker having a genetic disorder is...

	**9:** extremely more important than	**7:** very strongly more important than	**5:** strongly more important than	**3:** moderately more important than	**1:** equal to	**1/3:** moderately less important than	**1/5:** strongly less important than	**1/7:** very strongly less important than	**1/9:** extremely less important than
Timing of infection coincides with, or directly follows, other epidemic (ex. influenza, other CoVs)									
Amount of viral dose transmitted									
Other (*if you've specified an 'other' in Step 1*)									

If you have included 'other', please specify

Timing of infection coinciding with, or directly following, other epidemic (ex. influenza, other CoVs) is...

	**9:** extremely more important than	**7:** very strongly more important than	**5:** strongly more important than	**3:** moderately more important than	**1:** equal to	**1/3:** moderately less important than	**1/5:** strongly less important than	**1/7:** very strongly less important than	**1/9:** extremely less important than
Amount of viral dose transmitted	○	○	○	○	○	○	○	○	○
Other (*if you've specified an 'other' in Step 1*)	○	○	○	○	○	○	○	○	○

If you have included 'other', please specify

Amount of viral dose transmitted is...

	**9:** extremely more important than	**7:** very strongly more important than	**5:** strongly more important than	**3:** moderately more important than	**1:** equal to	**1/3:** moderately less important than	**1/5:** strongly less important than	**1/7:** very strongly less important than	**1/9:** extremely less important than
Other (*if you've specified an 'other' in Step 1*)	○	○	○	○	○	○	○	○	○

If you have included 'other', please specify

MERS Transmission Model: Expert Opinion Elicitation

Risk Factors and Pair-Wise Comparisons: Question 5

Please enter your general confidence level for the pairwise comparisons you did on the last page for Question 5, Step 2

	**1:** Completely unsure	**2:** Confident for a small part of answer only	**3:** Fairly confident; multiple doubts	**4:** Confident, with a few minor doubts	**5**: Very confident
Confidence level	○	○	○	○	○

Scenario 1: **ADULT DROMEDARY**

Imagine you are in the Arabian Peninsula.

Living in this place there are 10 men who all work with dromedary camels. They may work with the dromedaries in various ways: shepherds, camel farmers or farmhands, slaughterhouse workers, any other regular (such as daily) contact with dromedaries.

These camel-workers do not know eachother and do not cross-paths; they are independent and not working with the same dromedary herds.

Now, imagine that each one of these camel workers comes in contact with **one** infected MERS-CoV **ADULT** dromedary camel that is shedding virus.

What would be the minimum, maximum and most likely number of camel workers (**less than or equal to 50 years old**) who will become infected , if the type of regular contact with the infected dromedaries includes:

Options were numbers from 1-5

For all drop down menus here, options were all numbers from 0-10

	Minimum	Most likely	Maximum	Your Confidence Level (*1= not confident, 5=completely confident*)
Camel workers are regularly drinking **unpasteurised milk** from the MERS-CoV infected dromedaries				
Camel workers are regularly drinking/using **urine** from the MERS-CoV infected dromedaries				
Camel workers are regularly eating **raw meat** from the MERS-CoV infected dromedaries				
Camel workers have regular **direct, close contact** (ex. touching, kissing, assisting in births, milking, administering medicines, slaughtering) with infected dromedaries				
Camel workers have regular **indirect contact** (ex. cleaning animal housing area, handling animal waste) with infected dromedaries				

What would be the minimum, maximum and most likely number of camel workers (**greater than 50 years old**) who will become infected , if the type of regular contact with the infected dromedaries includes:

Options were numbers from 1-5

For all drop down menus here, options were all numbers from 0-10

	Minimum	Most likely	Maximum	Your Confidence Level (*1= not confident, 5=completely confident*)
Camel workers are regularly drinking **unpasteurised milk** from the MERS-CoV infected dromedaries				
Camel workers are regularly drinking/using **urine** from the MERS-CoV infected dromedaries				
Camel workers are regularly eating **raw meat** from the MERS-CoV infected dromedaries				
Camel workers have regular **direct, close contact** (ex. touching, kissing, assisting in births, milking, administering medicines, slaughtering) with infected dromedaries				
Camel workers have regular **indirect contact** (ex. cleaning animal housing area, handling animal waste) with infected dromedaries				

***Scenario 2:* JUVENILE DROMEDARY**

Imagine you are in the Arabian Peninsula.

Living in this place there are 10 men who all work with dromedary camels. They may work with the dromedaries in various ways: shepherds, camel farmers or farmhands, slaughterhouse workers, any other regular (such as daily) contact with dromedaries.

These camel-workers do not know eachother and do not cross-paths; they are independent and not working with the same dromedary herds.

Now, imagine that each one of these camel workers comes in contact with **one** infected MERS-CoV **JUVENILE** dromedary camel that is shedding virus.

What would be the minimum, maximum and most likely number of camel workers (**less than or equal to 50 years old**) who will become infected , if the type of regular contact with the infected dromedaries includes:

Options were numbers from 1-5

For all drop down menus here, options were all numbers from 0-10

	Minimum	Most likely	Maximum	Your Confidence Level (*1= not confident, 5=completely confident*)
Camel workers are drinking **unpasteurised milk** from the MERS-CoV infected dromedaries				
Camel workers are drinking/using **urine** from the MERS-CoV infected dromedaries				
Camel workers are eating **raw meat** from the MERS-CoV infected dromedaries				
Camel workers have **direct, close contact** (ex. touching, kissing, assisting in births, milking, administering medicines, slaughtering) with infected dromedaries				
Camel workers have **indirect contact** (ex. cleaning animal housing area, handling animal waste) with infected dromedaries				

What would be the minimum, maximum and most likely number of camel workers (**greater than 50 years old**) who will become infected , if the type of regular contact with the infected dromedaries includes:

Options were numbers from 1-5

For all drop down menus here, options were all numbers from 0-10

	Minimum	Most likely	Maximum	Your Confidence Level (*1= not confident, 5=completely confident*)
Camel workers are drinking **unpasteurised milk** from the MERS-CoV infected dromedaries				
Camel workers are drinking/using **urine** from the MERS-CoV infected dromedaries				
Camel workers are eating **raw meat** from the MERS-CoV infected dromedaries				
Camel workers have **direct, close contact** (ex. touching, kissing, assisting in births, milking, administering medicines, slaughtering) with infected dromedaries				
Camel workers have **indirect contact** (ex. cleaning animal housing area, handling animal waste) with infected dromedaries				

MERS Transmission Model: Expert Opinion Elicitation

Transmission Probabilities from Camels to Humans: Scenario 3

Scenario 3: CAMEL WORKERS TO CONTACTS

Imagine you are in the Arabian Peninsula.

Living in this place there are 10 men who all work with dromedary camels. They may work with the dromedaries in various ways: shepherds, camel farmers or farmhands, slaughterhouse workers, any other regular (such as daily) contact with dromedaries. Therefore, these men each have various types of contact with dromedaries, whether through ingesting camel products (ex. milk, urine, meat), through direct close contact (ex. touching, kissing, etc), or through indirect contact (ex. cleaning camel waste, living in camel environment).

Now, it turns out that at least one dromedary camel in each of the herds of these men is infected with MERS-CoV.

None of the camel workers have, as of yet, displayed any symptoms of illness, and therefore, we have **NOT done any laboratory testing to confirm** whether or not they are MERS-CoV positive.

Now, imagine that each of these camel workers goes home, every evening, to live, eat, and sleep in a closed quarters/house where there is one (non-camel working) family member.

What would be the minimum, maximum and most likely number of<u>family members</u> who may become infected?

Options were numbers from 1-5

For all drop down menus here, options were all numbers from 0-10

	Minimum	Most likely	Maximum	Your Confidence Level (*1= not confident, 5=completely confident*)
Camel workers are less than or equal to 50 years old				
Camel workers are greater than 50 years old				

MERS Transmission Model: Expert Opinion Elicitation

Open Ended Questions

Question 1:

Are there any factors that you believe may lead to **increased viral shedding** in MERS-CoV infected camels?

Please enter your confidence level for your response to Question 1

	**1:** Completely unsure	**2:** Confident for a small part of answer only	**3:** Fairly confident; multiple doubts	**4:** Confident, with a few minor doubts	**5**: Very confident
Confidence level	○	○	○	○	○

Question 2:

Are there any other factors that you believe may lead to increased/more efficient transmission between MERS-CoV infected camels and humans? (*Note: if you do not believe that camels play any role in human infection, please indicate so in the text place below and then skip this question***).**

Please enter your confidence level for your response to Question 1

	**1:** Completely unsure	**2:** Confident for a small part of answer only	**3:** Fairly confident; multiple doubts	**4:** Confident, with a few minor doubts	**5**: Very confident
Confidence level	○	○	○	○	○

Question 3:

Do you think there are any other viruses which may cross-immunise with MERS-CoV in either camels or humans?

Please enter your confidence level for your response to Question 2

	**1:** Completely unsure	**2:** Confident for a small part of answer only	**3:** Fairly confident; multiple doubts	**4:** Confident, with a few minor doubts	**5**: Very confident
Confidence level	○	○	○	○	○

MERS Transmission Model: Expert Opinion Elicitation

Comments on the Questionnaire

Is there any important risk factor, transmission pathway, or other element that you feel we have missed in this questionnaire? Please specify...

Do you have any other comment on your experience doing the questionnaire or things we should consider when analysing your answers? Please specify...

Can you recommend another expert in this field who you feel would be useful to have fill out the questionnaire? If so, please give their details below so that we can contact them...

Name

Affiliation/Organisation

City/Town

Country

Email Address

MERS Transmission Model: Expert Opinion Elicitation

Thank you!

Please press the "**Finish**" button below to finalise the questionnaire once you are sure you have finished. If you need to go back to change or check any of your answers, you are able to do so using the **"Back"** button. Once you press the **"Finish"** button, the questionnaire will be sent to us automatically.

If you have any comment you would like to make, or change to your answers after you have sent the questionnaire, you are welcome to do so by *contacting Anna Funk at anna-louise.funk@pasteur.fr*

We will aim to follow-up with you with a summary of all expert answers in the next week or two.

Thank you for sharing your expertise and your time!
